# EYA2 suppresses the progression of hepatocellular carcinoma via SOCS3-mediated blockade of JAK/STAT signaling

**DOI:** 10.1186/s12943-021-01377-9

**Published:** 2021-05-27

**Authors:** Ze-Kun Liu, Can Li, Ren-Yu Zhang, Ding Wei, Yu-Kui Shang, Yu-Le Yong, Ling-Min Kong, Nai-Shan Zheng, Ke Liu, Meng Lu, Man Liu, Cai-Xia Hu, Xiao-Zhen Yang, Zhi-Nan Chen, Huijie Bian

**Affiliations:** 1 National Translational Science Center for Molecular Medicine, Department of Cell Biology, State Key Laboratory of Cancer Biology, Fourth Military Medical University, Xi’an, 710032 China; 2 School of Life Sciences, Central China Normal University, Wuhan, 430079 China; 3 Oncology and Hepatobiliary Minimally Invasive Interventional Center, Beijing Youan Hospital, Capital Medical University, Beijing, 100069 China

**Keywords:** Whole-exome sequencing, Eyes absent homolog 2, Somatic mutation, Tumor suppressor gene, Unfolded protein response, JAK/STAT signaling pathway

## Abstract

**Background:**

Somatic mutations are involved in hepatocellular carcinoma (HCC) progression, but the genetic mechanism associated to hepatocarcinogenesis remains poorly understood. We report that Eyes absent homolog 2 (EYA2) suppresses the HCC progression, while EYA2(A510E) mutation identified by exome sequencing attenuates the tumor-inhibiting effect of EYA2.

**Methods:**

Whole-exome sequencing was performed on six pairs of human HCC primary tumors and matched adjacent tissues. Focusing on EYA2, expression level of EYA2 in human HCC samples was evaluated by quantitative real-time PCR, western blot and immunohistochemistry. Loss- and gain-of-function studies, hepatocyte-specific deletion of EYA2 (*Eya2*^−/−^) in mice and RNA sequencing analysis were used to explore the functional effect and mechanism of EYA2 on HCC cell growth and metastasis. EYA2 methylation status was evaluated using Sequenom MassARRAY and publicly available data analysis.

**Results:**

A new somatic mutation p.Ala510Glu of EYA2 was identified in HCC tissues. The expression of EYA2 was down-regulated in HCC and associated with tumor size (*P* = 0.001), Barcelona Clinic Liver Cancer stage (*P* = 0.016) and tumor differentiation (*P* = 0.048). High level of EYA2 was correlated with a favorable prognosis in HCC patients (*P* = 0.003). Results from loss-of-function and gain-of-function experiments suggested that knockdown of EYA2 enhanced, while overexpression of EYA2 attenuated, the proliferation, clone formation, invasion, and migration of HCC cells in vitro. Delivery of EYA2 gene had a therapeutic effect on inhibition of orthotopic liver tumor in nude mice. However, EYA2(A510E) mutation led to protein degradation by unfolded protein response, thus weakening the inhibitory function of EYA2. Hepatocyte-specific deletion of EYA2 in mice dramatically promoted diethylnitrosamine-induced HCC development. EYA2 was also down-regulated in HCC by aberrant CpG methylation. Mechanically, EYA2 combined with DACH1 to transcriptionally regulate SOCS3 expression, thus suppressing the progression of HCC via SOCS3-mediated blockade of the JAK/STAT signaling pathway.

**Conclusions:**

In our study, we identified and validated EYA2 as a tumor suppressor gene in HCC, providing a new insight into HCC pathogenesis.

**Supplementary Information:**

The online version contains supplementary material available at 10.1186/s12943-021-01377-9.

## Introduction

The progression in understanding the molecular basis of hepatocarcinogenesis has attributed to genetic and epigenetic analyses of oncogenes and tumor-suppressor genes [1]. In general view, the genesis of cancer can be triggered by loss-of-function mutations in tumor suppressor genes and gain-of-function mutations in proto-oncogenes [2]. In addition, epigenetic alterations on the genome can result in the abnormal transcription, and further contribute to the malignant transformation of tumors [2]. Although great progress in surgical resection, liver transplantation and therapy using several drugs including sorafenib, regorafenib and nivolumab significantly improved the prognosis of patients with hepatocellular carcinoma (HCC) [3], and the high heterogeneity of liver cancer, the postoperative recurrence and drug resistance are affecting the prognosis. Despite the improvements in the knowledge of the characteristic of this type of tumor, the pathogenesis of HCC is still unclear, and specific targeted drugs are still lacking. Thus, the discovery of additional genes potentially regulating HCC might provide important suggestions in the development of new drugs and clinical treatments.

Eyes absent homolog (EYA) is a transcriptional co-activator with a phosphatase function characterized by the presence of 271 amino acid residues in the C-terminal domain that encodes a highly conserved domain known as the ED domain. EYA can interact with proteins such as DACH (Dachshund) and SIX (Sine Oculis Homeobox) through its conserved ED domain to form a transcription complex that translocates into the nucleus to control cell proliferation and DNA damage repair in development and disease [4]. On the other hand, EYA can be phosphorylated by the Abelson tyrosine kinase, which recruits EYA to the cytoplasm to regulate cell polarity and innate immunity [4, 5]. EYA2 is a member of the EYA protein family, and it is characterized by a tissue specific expression in different types of tumors in which plays contrary functions. For example, EYA2 can form a complex with SIX1 to regulate epithelial-mesenchymal transition by the activation of TGF-β pathway, consequently promoting the metastasis of breast cancer cells [6]. Moreover, EYA2 promotes tumor progression in other tumors, including lung [7] and osteosarcoma [8]. On the other hand, a stable knockdown of EYA2 increases cell proliferation and metastasis of pancreatic adenocarcinoma [9]. However, the direct effects of EYA2 on hepatocarcinogenesis and the clinical significance of the expression of EYA2 in patients with HCC are largely unknown.

In this work we performed whole-exome sequencing on six pairs of HCC tissues and case-matched adjacent tissues clinically and pathologically characterized to identify new somatic mutant genes. Our results identified EYA2 as a potential suppressor gene in HCC, while EYA2(A510E) mutant type reduced its suppressing function. The overexpression of EYA2 predicted a favorable prognosis in patients with HCC. Moreover, using hepatocyte-specific EYA2-knockout mice (*Eya2*^−/−^), we demonstrated that EYA2 acted as a tumor suppressor in the occurrence and progression of HCC through SOCS3-mediated blockade of JAK/STAT signaling.

## Materials and methods

### Liver tissues from patients with HCC

We obtained six pairs of HCC and adjacent tissues by direct puncture from the Youan Hospital, Capital Medical University of China (Beijing, China) that we used for whole-exome sequencing. All patients did not receive any chemotherapy or radiotherapy prior to resection. In addition, we obtained 94 paired HCC and adjacent tissues from the Xijing Hospital of Fourth Military Medical University (Xi’an, China). Each patient provided a written informed consent and we obtained the ethical approval from the Ethical Committee and Institutional Review Board of Fourth Military Medical University.

### Animal studies

All animal experiments were performed in accordance with ethical guidelines on animal care, and as directed by the Animal Care Committee of the Fourth Military Medical University.

### Statistical methods

All data were obtained from three independent experiments and analyzed as the means ± SEM using GraphPad Prism v5.01 software (GraphPad Software, CA, USA) and SPSS 19.0 (SPSS, Inc., IL, USA). Two-tailed Student’s *t*-tests (two-sample equal variance) were used to test the significance of differences between two groups. Pearson’s correlation test was used to assess the correlation between EYA2 mRNA expression and its methylation, as well as the correlation between EYA2 mRNA expression and other genes, such as DACH1 and SOCS3 expression. Spearman’s correlation was used to analyze the correlation between EYA2 and SOCS3 expression by immunohistochemistry. The association between the expression of EYA2 and clinical pathologic variables were computed using chi-square test, while Kaplan-Meier survival analysis was used to compare HCC patient survival by the log rank test. Cox proportional hazards regression analyses were performed to evaluate the effect of clinical variables on HCC patient survival. *P* < 0.05 was considered statistically significant.

Additional detailed materials and methods can be found in Additional file 1: Supplementary materials and methods.

## Results

### EYA2 somatic mutations in HCC are identified by exome sequencing analysis

The genomic DNA obtained from six pairs of HCC primary tumors and matched adjacent tissues was analyzed by whole-exome sequencing. The clinical characteristics of the patients with HCC are shown in (Additional file 2: Table S1). After the removal of duplicates and low-quality reads, a mean coverage depth of 78.82-fold for HCC and 78.29-fold for paired adjacent tissues was achieved, and more than 77.09% of target exome was covered by at least 20 × (Additional file 2: Table S2). A three-caller bioinformatics pipeline, previously designed by our laboratory [10], was conducted to identify the somatic variants (SNVs and indels) by comparing variants identified in the HCC and the tumor exome datasets, against dbSNP and germline variants which were presented in the matched adjacent samples. As shown in Additional file 3: Fig. S1, 271 somatic mutations in the HCC tumors, including 189 somatic non-silent mutations and 20 indels were identified. A total of 209 nonsynonymous somatic variants were observed within 203 genes (Additional file 2: Table S3). The average frequency of nonsynonymous variants was 34.8 per affected individual and the nonsynonymous to synonymous somatic SNV ratio was 3.39 (Additional file 2: Table S4). We found that the mutation types and prevalence were similar to that of previously reported HBV-associated HCC genome [11].

**Fig. 1:**
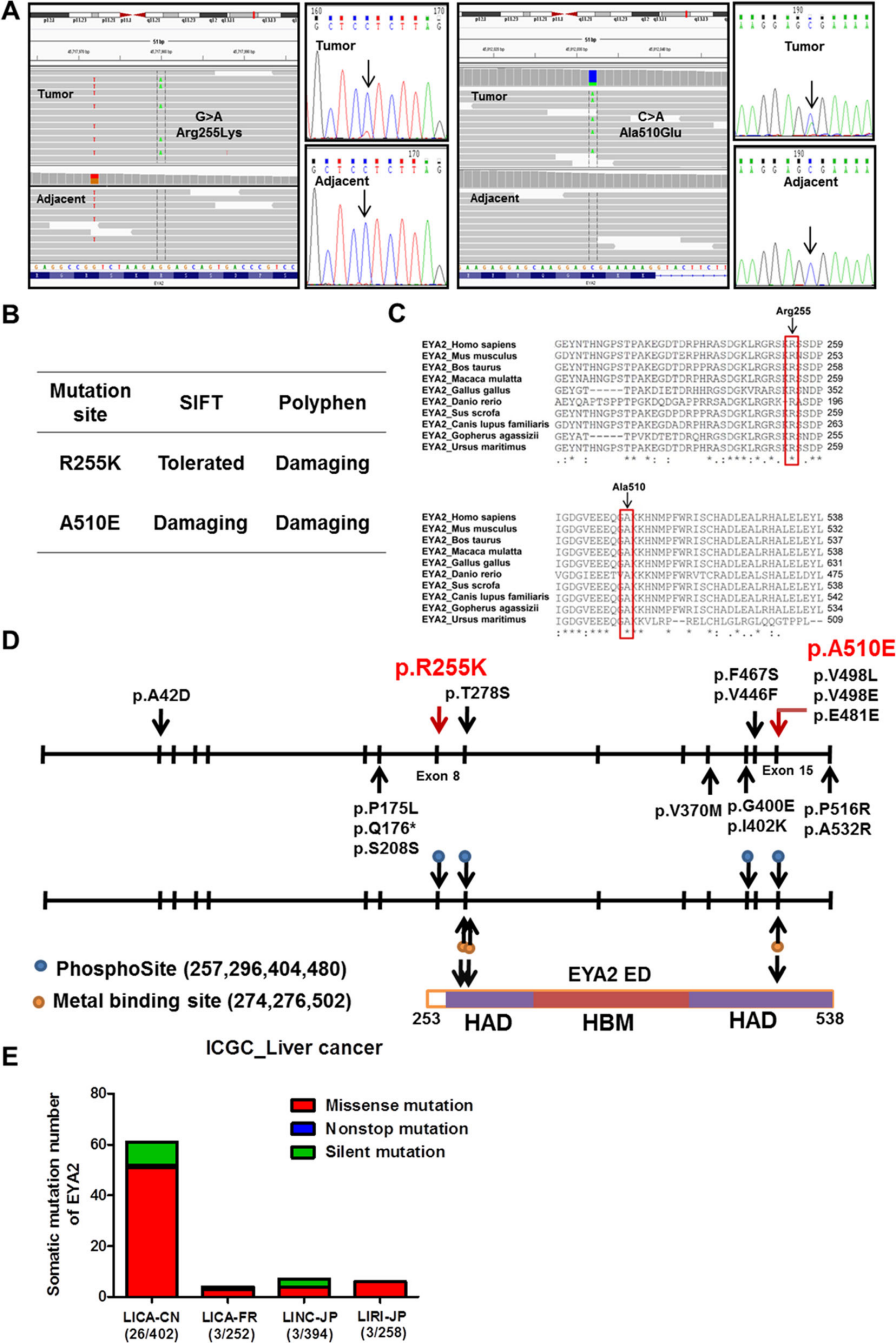
EYA2 mutations in HCC. (A) Somatic mutations identified in EYA2 gene, which were confirmed by IGV visualization and Sanger sequencing of DNA samples from HCC and case-matched adjacent tissues. The black arrows indicate the somatic mutation sites. (B) Prediction of the functional effects of R255K and A510E substitutions (R, Arg; K, Lys; A, Ala; E, Glu). (C) Multiple sequence alignments of EYA2 paralogs from different species. The mutation sites in EYA2 are indicated by black arrows. (D) Genomic organization of EYA2 exon regions and protein domain structure. The EYA2 somatic mutations in HCC are indicated by red and black arrows which represent our research findings and COSMIC database, respectively. The asterisk represents nonsense mutation. (E) Somatic mutation frequency of EYA2 in liver cancer in the population of different countries obtained from the ICGC database

Next, we selected genes somatically mutated in two or more tumors in the discovery set for further study [12], or multiple somatic mutations in the specific identical gene in the same sample. The totally six genes were identified, including three non-silent mutations EYA2, GPR98 and UBE2S, in which the somatic mutation gene EYA2 had a highest allelic fraction (Additional file 3: Fig. S2). Two new mutations p.R255K and p.A510E of EYA2 gene, which are not yet reported in HCC, were identified in two of the six tumors (Fig. 1A). Both alterations were confirmed using IGV visualization and PCR-based Sanger sequencing of HCC and matched adjacent tissues from the two patients (Fig. 1A). Both mutations were heterozygous and predicted to be potentially deleterious (Fig. 1A, B). We found that the Arg255 and Ala510 residues were highly conserved through evolution by aligning the sequences of homologous EYA2 among different species (Fig. 1C). The somatic mutations of EYA2 in HCC reported in the COSMIC database and the literatures and disclosed in this study are summarized in Fig. 1D, which is showing that the Arg255Lys and Ala510Glu were generated by missense mutations in the highly conservative ED domain region. Moreover, we analyzed 61 somatic mutations of EYA2 in 26 of the 402 Chinese patients with liver cancer using the ICGC database, thus demonstrating a mutation frequency of 6.47%, which was higher than that in other countries (Fig. 1E). These data show that somatic variants of EYA2 gene may be of great significance in Chinese patients.

### EYA2 is down-regulated in HCC and correlated with aggressive tumor progression and poor prognosis in patients with HCC

**Fig. 2:**
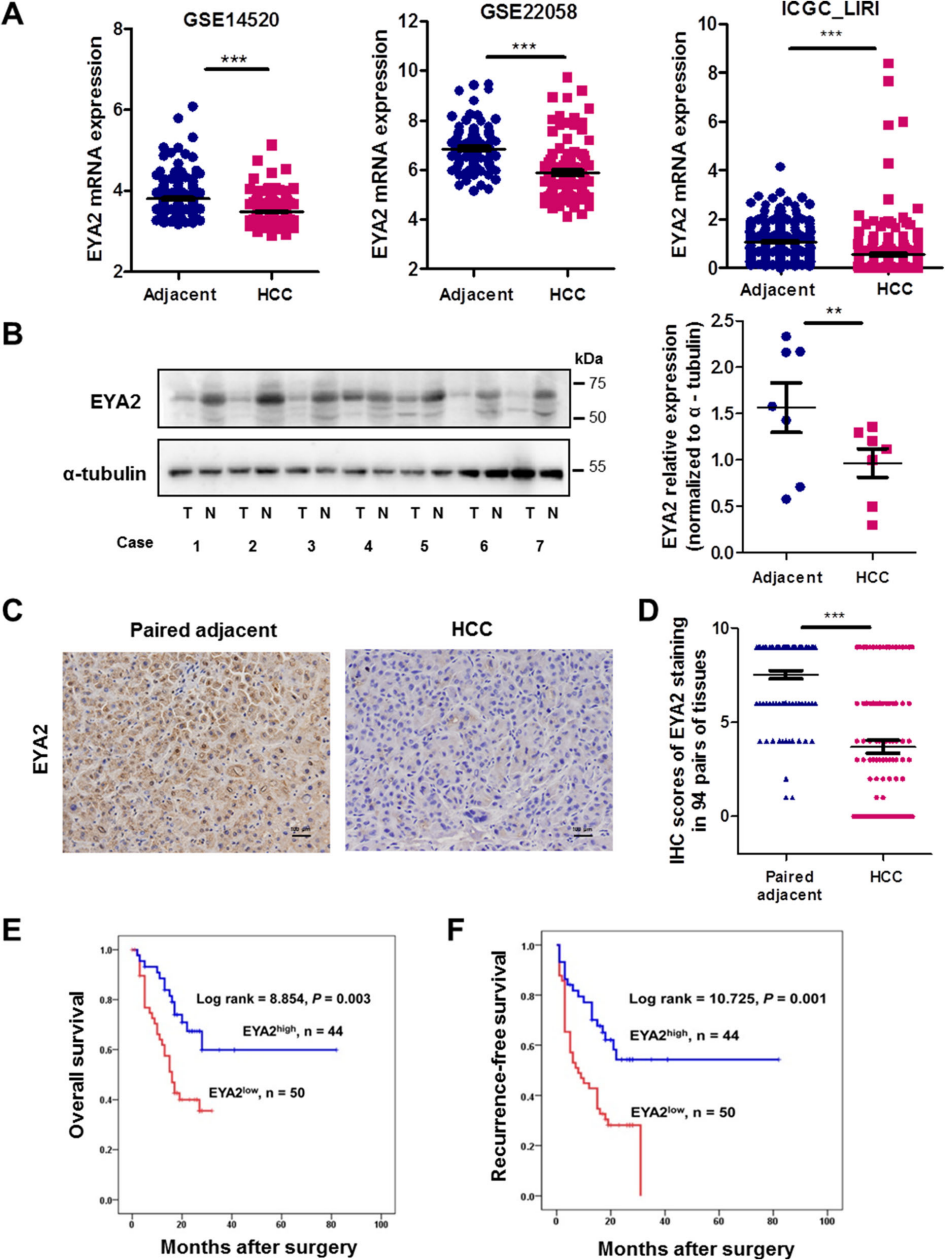
Expression and prognostic significance of EYA2 in HCC tissues. (A) EYA2 expression in HCC and adjacent tissues according to the three datasets GES22058, GSE14520 and ICGC_LIRI. (B) Western blot analysis of EYA2 expression in HCC and adjacent tissues. T: HCC tissues, N: Adjacent tissues. (C) A representative immunohistochemical staining of EYA2 in HCC and case-matched adjacent tissue. (D) Immunohistochemistry scores associated to the EYA2 expression in HCC and case-matched adjacent tissues (*n =* 94 pairs). Kaplan-Meier analysis of overall survival (E) and recurrence-free survival (F) of HCC patients with high or low EYA2 expression (*n =* 94 cases). **P < 0.01, ***P < 0.001. (A, B, D) mean ± SEM, Student’s *t*-tests; (E, F) Kaplan-Meier analysis

TCGA pan-cancer analysis revealed that the EYA2 expression was significantly increased in cancer, including GBM, LUSC and UCEC, compared with the respective adjacent tissues (Additional file 3: Fig. S3A). However, EYA2 expression was significantly declined in BRCA, COAD, HNSC, KICH, KIRC, KIRP, LIHC, READ, STAD and THCA than that in their respective adjacent tissues (Additional file 3: Fig. S3A). EYA2 was expressed at a low level in liver cancer cells compared to its expression in a wide range of cancer cells by CCLE database (Additional file 3: Fig. S3B). Furthermore, we analyzed EYA2 expression in HCC and adjacent non-tumor tissues using other three datasets downloaded from GSE22058, GSE14520 and ICGC_LIRI, which showed that the EYA2 mRNA expression was decreased in HCC compared with adjacent tissues (Fig. 2A).

We selected seven pairs of HCC and matched adjacent tissues to explore EYA2 protein expression, which showed a lower expression of EYA2 in HCC than that in the adjacent tissues (Fig. 2B). Furthermore, we performed immunohistochemistry to detect the EYA2 expression in a cohort of 94 pairs of HCC and matched adjacent tissues. The clinical information of the patients with HCC is shown in (Additional file 2: Table S5). Consistently, the EYA2 expression was significantly decreased in HCC compared with its expression in the paired adjacent tissues (Fig. 2C, D). Furthermore, EYA2 expression in adjacent tissues had an apparent nuclear and cytoplasm localization (Fig. 2C). The analysis of the correlation between EYA2 expression and clinical pathological features revealed that the EYA2^high^ phenotype was significantly associated with tumor size less than 5 cm (*P* = 0.001), Barcelona Clinic Liver Cancer (BCLC) stage with 0–A (*P* = 0.016) and tumor differentiation with I–II (*P* = 0.048) (Table 1). The univariate analysis revealed that the EYA2 expression was significantly associated with the overall survival (Additional file 2: Table S6). We also performed a multivariate Cox regression analysis to further explain the prognostic significance of EYA2 expression using all the variables that were identified as significant by the univariate analysis. Multivariate analysis revealed that the serum α-fetoprotein (*P* = 0.030), tumor differentiation (*P* = 0.040) and EYA2 protein expression (*P* = 0.042) were independent prognostic factors for the overall survival (Additional file 2: Table S6). The Kaplan-Meier analysis indicated that the high EYA2 expression resulted in a significantly more favorable prognosis (Fig. 2E) and a lower cumulative recurrence rate (Fig. 2F). These data indicate that down-regulation of EYA2 contributes to HCC malignant progression.

**Table 1:** Clinical features and EYA2 protein expression in patients with HCC (*n* = 94)

Clinicopathological	EYA2 protein expression		*P* value
variables	High (*n* = 44, 46.81%)	Low (*n* = 50, 53.19%)	
Age (years)			
≥ 50	31 (32.98%)	27 (28.72%)	0.102
< 50	13 (13.83%)	23 (24.47%)	
Gender			
Male	39 (41.49%)	43 (45.74%)	0.702
Female	5 (5.32%)	7 (7.45%)	
Tumor number			
single	32 (34.04%)	37 (39.36%)	0.889
multiple	12 (12.77%)	13 (13.83%)	
Tumor size (cm)			
< 5	24 (25.53%)	11 (11.70%)	0.001
≥ 5	20 (21.28%)	39 (41.49%)	
Liver cirrhosis			
Yes	31 (32.98%)	30 (31.91%)	0.289
No	13 (13.83%)	20 (21.28%)	
Serum AFP (ng/ml)			
≤ 20	19 (20.21%)	14 (14.89%)	0.124
> 20	25 (26.60%)	36 (38.30%)	
HBsAg			
Positive	41 (43.62%)	43 (45.74%)	0.429
Negative	3 (3.19%)	7 (7.45%)	
BCLC stage			
0–A	23 (24.47%)	14 (14.89%)	0.016
B–C	21 (22.34%)	36 (38.30%)	
TNM stage			
I	30 (31.91%)	28 (29.79%)	0.225
II–IV	14 (14.89%)	22 (23.40%)	
Tumor differentiation			
I–II	21 (22.34%)	14 (14.89%)	0.048
III–IV	23 (24.47%)	36 (38.30%)	

Chi-square test was performed for statistical analysis. *P* < 0.05 was considered statistically significant. *AFP* α-fetoprotein, *HBsAg* hepatitis B surface antigen, *BCLC* Barcelona Clinic Liver Cancer, *TNM* tumor node metastasis

### EYA2 inhibits the malignant phenotype of HCC cells which is diminished by EYA2(A510E) mutant type

**Fig. 3:**
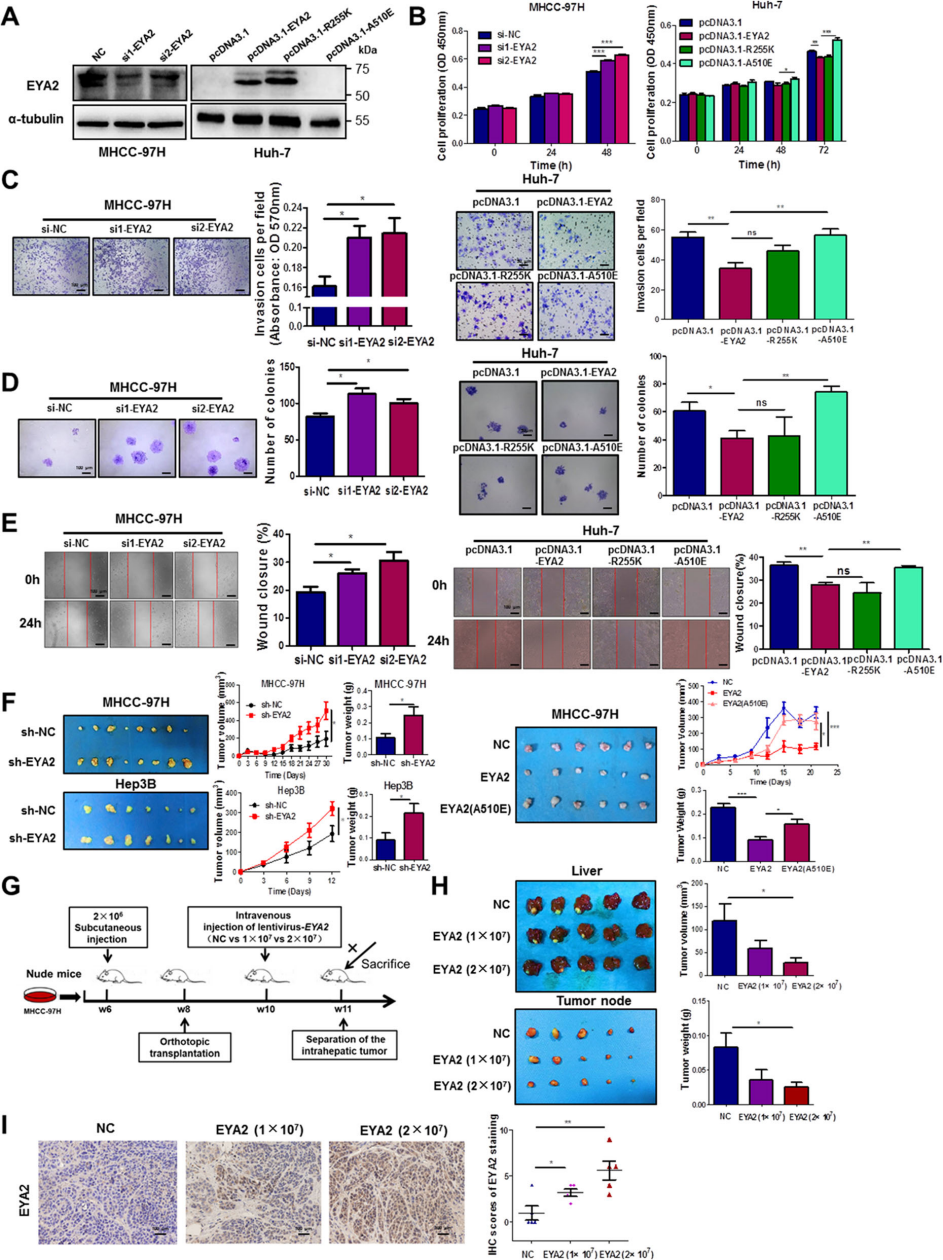
EYA2 suppresses the malignant phenotypes of HCC cells in vitro and in vivo. (A) Western blot analysis of the expression of EYA2 in HCC cells transiently transfected with siRNA or with the overexpression and mutant vectors. The effect of siRNA-mediated knockdown of EYA2 and pcDNA3.1-mediated overexpression of constructs encoding EYA2 wild-type, EYA2(R255K) and EYA2(A510E) on cell proliferation (B), cell invasion (C), clone formation (D) and cell migration (E) of HCC cells in vitro. (F) The effect of EYA2 knockdown, EYA2 wild-type and EYA2(A510E) mutant on HCC tumor growth in nude mice. (G) The scheme of treatment of orthotopic HCC tumors in nude mice by lentivirus-EYA2. (H) Images, volume and weight of the orthotopic liver tumors of the mice. (I) Expression of EYA2 in tumor tissues detected by immunohistochemistry analysis. ns, not significant, *P < 0.05, **P < 0.01, ***P < 0.001. (B–F, H–I) mean ± SEM, Student’s *t*-tests

Our results showed that overexpression of EYA2 promoted the proliferation and invasion of breast cancer cells, MCF-7 and lung cancer cells, A549 cells, and revealed a certain inhibitory effect on colon cancer cells, HT-29 and pancreatic cancer cells, PANC-1 cells (Additional file 3: Fig. S3C–E), which was consistent with the previous studies by others [6, 7, 9]. To assess the role of EYA2 in HCC, EYA2 expression in five HCC cell lines were checked (Additional file 3: Fig. S4A). Overexpression and knockdown performances were conducted in Huh-7 and MHCC-97H cell lines, respectively (Fig. 3A). Knockdown of EYA2 significantly promoted the cell proliferation, invasion, clone formation and migration in vitro, while overexpression of EYA2 inhibited those behaviors (Fig. 3B–E). Interestingly, the A510E mutant type reduced the inhibitory function of wild-type EYA2 on the biological behavior of Huh-7 cells, while R255K mutant type had no significant effect (Fig. 3B–E). Furthermore, we established a stable EYA2 knockdown in MHCC-97H and Hep3B cell lines (Additional file 3: Fig. S4B,C), and a stable overexpression of EYA2 in MHCC-97H cell line (Additional file 3: Fig. S4C), and obtained the consistent results (Additional file 3: Fig. S4D,E). Cell cycle analysis indicated that the knockdown of EYA2 promoted G_1_/S phase transition in HCC cells (Additional file 3: Fig. S4F). The apoptosis assay showed that the percentage of apoptotic cells significantly reduced in HCC cells with EYA2 knockdown (Additional file 3: Fig. S4G). Western blot analysis revealed that the overexpression of EYA2 resulted in a higher expression of P27 and BAX and a lower expression of cyclin D1 and CDK2 compared to their expression in the negative control group, which was observed opposite in Hep3B-sh-EYA2 cells (Additional file 3: Fig. S4H).

We next assessed the effect of EYA2 on the tumorigenicity of HCC cells in vivo. MHCC-97H and Hep3B cells with stable EYA2 knockdown were subcutaneously injected into the flank of nude mice. As shown in Fig. 3F, EYA2 knockdown significantly increased tumor growth and tumor weight. In contrast, EYA2 overexpression significantly reduced the subcutaneously tumorigenic ability of the tumor cells in nude mice, and EYA2(A510E) mutation dispelled the inhibitory effect (Fig. 3F).

Finally, we evaluated the inhibitory effect of EYA2 on orthotopic liver cancer transplantation in a nude mouse model. The 2 × 10^7^ TU lentivirus carrying the EYA2 gene that was injected into the tail vein of nude mice had a good inhibitory effect on the tumor growth in situ. The tumor volume (*P* = 0.0398) and tumor weight (*P* = 0.0368) in the 2 × 10^7^ TU lentivirus-EYA2 group were significantly reduced compared with those in the control group (Fig. 3G, H). In addition, immunohistochemistry and western blot analyses showed that the expression of EYA2 in the tumor tissues of the lentivirus-EYA2 group was significantly higher than that of control group (Fig. 3I and Additional file 3: Fig. S5). Taken together, these data demonstrate that EYA2 acts as a tumor suppressor to prevent the initiation and progression of HCC, while EYA2(A510E) mutant type attenuates the inhibitory function.

### EYA2 is frequently low-expressed in HCC by aberrant CpG methylation

**Fig. 4:**
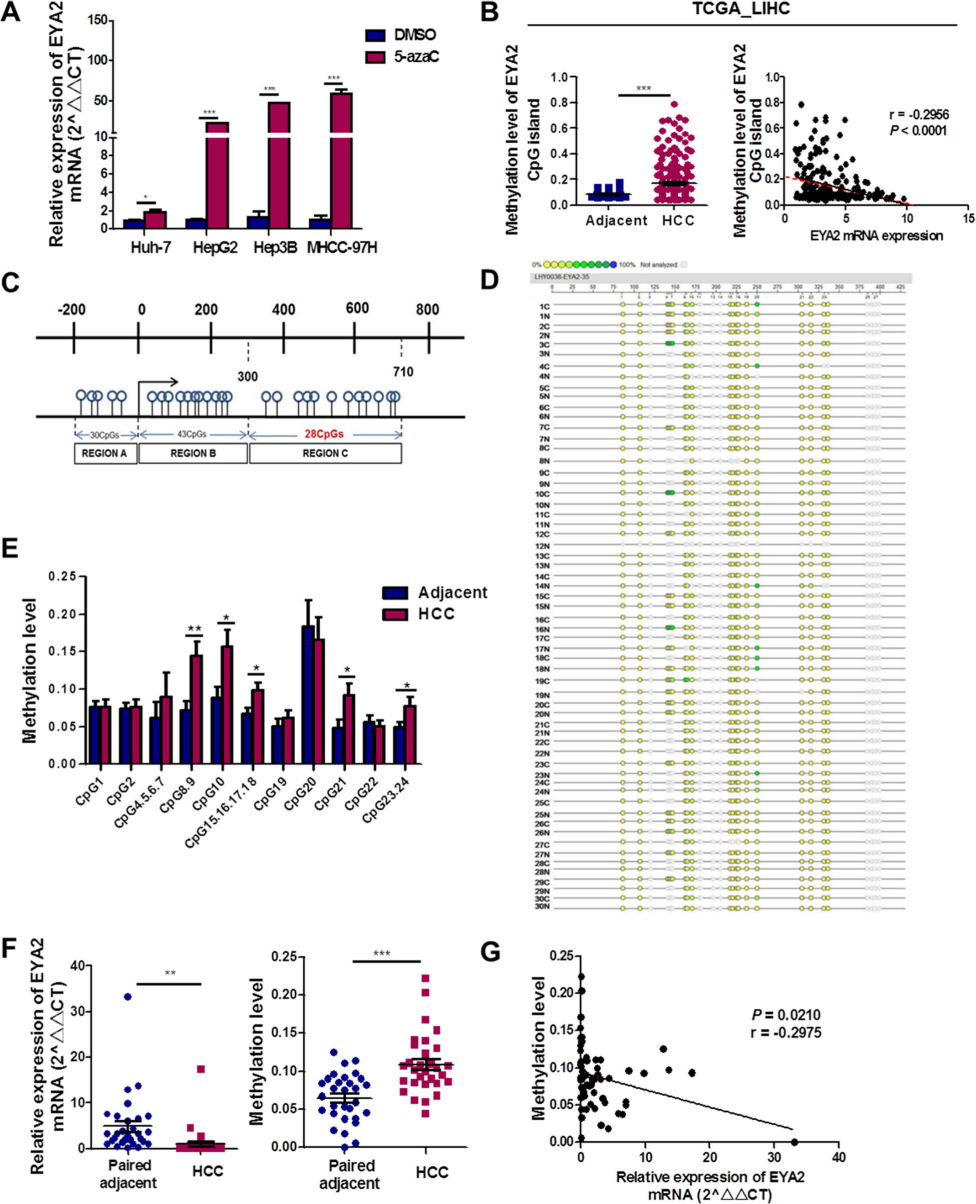
Aberrant CpG methylation of the first intron region of EYA2 down-regulates the expression of mRNA in HCC. (A) mRNA expression of EYA2 in four HCC cell lines by qRT-PCR after treatment with 5-aza-2′-deoxycytidine for 12 h compared with DMSO-treated cells. (B) The methylation level of EYA2 CpG islands in HCC and adjacent tissue (left), and negative correlation between methylation level of EYA2 CpG islands and EYA2 mRNA expression based on MethHC database (right). (C) The location of CpG in EYA2 intron 1 and PCR primers used for the methylation analysis. (D) Profiling of the methylation levels of CpG sites in EYA2 intron 1 presented as a dot chart. (E) Average methylation level of each CpG site in HCC and adjacent tissues (*n* = 30 pairs). (F) mRNA expression of EYA2 (left) and 10 significantly different CpG methylation sites (right) in 30 paired HCC tissues and adjacent tissues. (G) Correlation analysis between mean methylation of 10 significantly different CpGs and corresponding mRNA expression of EYA2. *P < 0.05, **P < 0.01, ***P < 0.001. (A, B, E, F) mean ± SEM, Student’s *t*-tests; (B, G) Pearson’s correlation test

To further explore the methylation status near the EYA2 transcription start site, we treated four liver cancer cell lines with the demethylating agent 5-aza-2′-deoxycytidine (5-azaC). As shown in Fig. 4A, 5-azaC significantly increased the EYA2 mRNA expression. The region near the EYA2 transcription start site was analyzed to predict the CpG islands (Additional file 3: Fig. S6A). The methylation level of EYA2 CpG islands in HCC was evaluated using MethHC database. EYA2 CpG islands were hypermethylated in HCC than that in adjacent tissues and negative correlation between methylation level of EYA2 CpG islands and EYA2 mRNA expression (Fig. 4B). We next identified the methylated region in the intron 1 of EYA2, which was 411 bp long and included 28 CpGs (Fig. 4C), by Sequenom EpiTyper MassARRAY in 30 paired HCC and the matched adjacent tissues. The results demonstrated that 10 of 28 CpG sites located in the intron 1 of EYA2 were hypermethylated in HCC compared with the same intron in the matched adjacent tissues, and consequently the mRNA expression in the corresponding HCC tissues was significantly lower than that in the matched adjacent tissues (Fig. 4D–F, Additional file 2: Table S7). Furthermore, the mean methylation of 10 CpGs was negatively correlated with the mRNA expression of EYA2 (Fig. 4G).

We also investigated the methylation status of EYA2 in all the 13 cancer types available in the TCGA pan-cancer database, and we discovered that seven types of tumor (BRCA, COAD, HNSC, KIRC, KIRP, LUAD and PRAD) were characterized by the hypermethylation of EYA2, while others (BLCA and UCEC) were characterized by its hypomethylation (Additional file 3: Fig. S6B). Except for ESCA, KIRC, BLCA and PAAD, all the other nine tumors showed a negative correlation between methylation level and mRNA expression of EYA2 (Additional file 3: Fig. S6C). However, we did not find any significant association between EYA2 expression and EYA2 copy number variants in HCC (Additional file 3: Fig. S6D,E). These results indicate that DNA methylation of EYA2 locus is involved in the decreased EYA2 expression in HCC.

### EYA2(A510E) mutation leads to protein degradation by unfolded protein response via lysosome pathway and ubiquitination-independent proteasome pathway

In this study, EYA2(A510E) mutation led to a loss of function of wild-type EYA2 in HCC (Fig. 3B–F; Additional file 3: Fig. S4D,E). We transiently transfected EYA2 wild-type or EYA2(A510E) mutant in HEK293T and Huh-7 cell lines. qRT-PCR and western blot analyses showed that the EYA2 mRNA expression was not decreased in the A510E mutant group, but its protein expression was markedly reduced (Additional file 3: Fig. S7A,B; Fig. 3A). The results were confirmed in HEK293T, Huh-7 and MHCC-97H cells with stable expression of EYA2 wild-type or EYA2(A510E) mutant (Additional file 3: Fig. S7C,D and Fig. S4C).

**Fig. 5:**
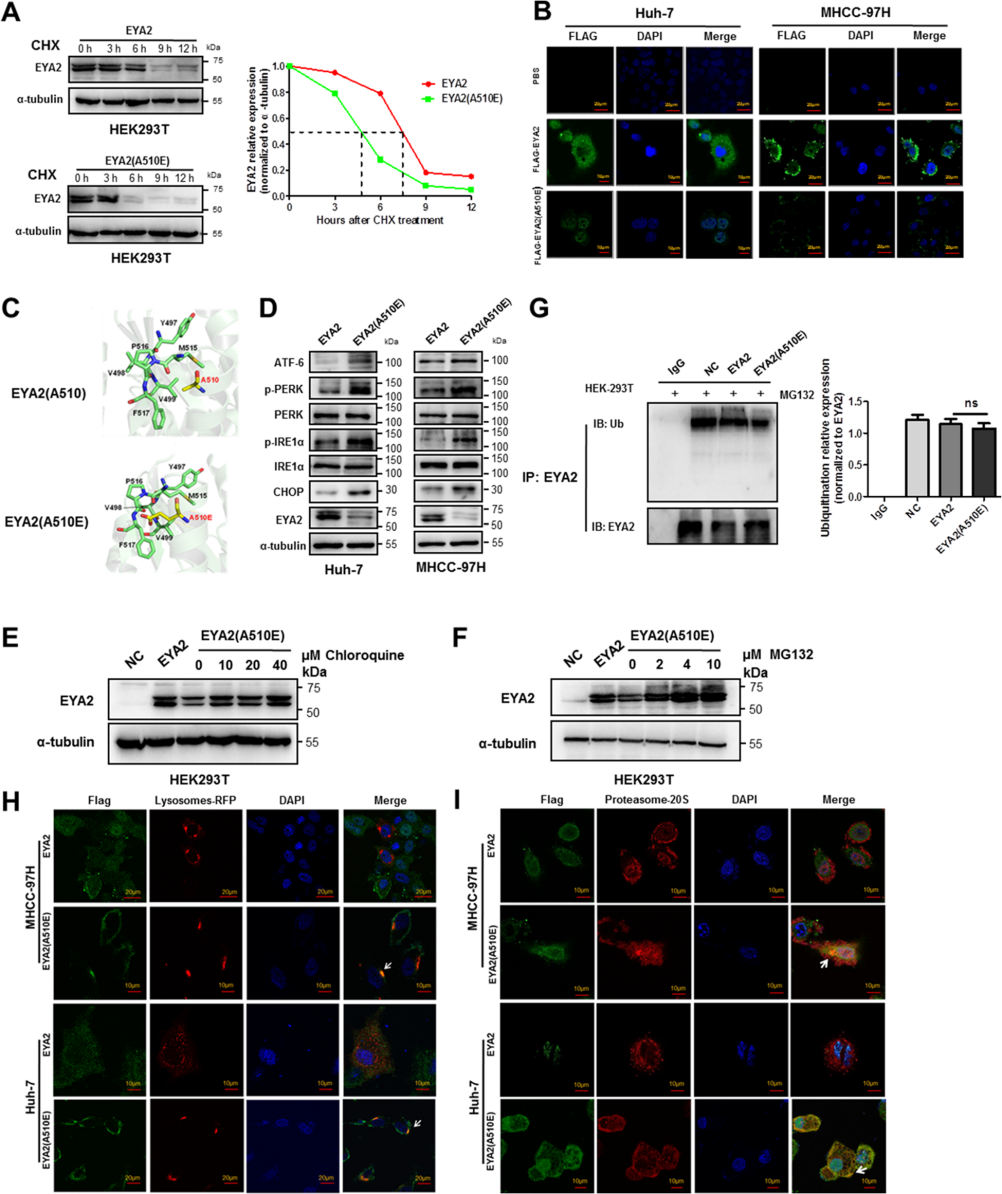
EYA2(A510E) mutation leads to lysosomal or proteasomal degradation by triggering the unfolded protein response. (A) EYA2(A510E) shortened the half-life of EYA2 protein. EYA2 and EYA2(A510E) stably transfected HEK293T cells were treated with 100 μg/ml cycloheximide (CHX) and protein lysates were collected at the indicated times for western blot analysis. (B) Confocal immunofluorescence analysis of the expression of Flag-tagged EYA2(A510E) and Flag-tagged EYA2. (C) Structural prediction of EYA2(A510E) mutation. (D) Western blot analysis of the expression of UPR-related proteins in EYA2(A510E) mutant-expressed Huh-7 and MHCC-97H cells. Western blot analysis of EYA2 expression in EYA2(A510E) stably transfected HEK293T cells treated with chloroquine (E) or MG132 (F). (G) Immunoprecipitation analysis of the ubiquitination of EYA2(A510E) mutation. Western blot densitometry was performed from three independent experiments. EYA2 was used as the normalization control. (H) Colocalization of Flag-tagged EYA2(A510E) and lysosomes in the presence of chloroquine detected by confocal immunofluorescence. The arrows point the colocalization of Flag-tagged EYA2(A510E) and Lysosomes-RFP. (I) Colocalization of Flag-tagged EYA2(A510E) and proteasome-20S in the presence of MG132 detected by confocal immunofluorescence. The arrows point the colocalization of Flag-tagged EYA2(A510E) and proteasome-20S. ns: not significant. (G) mean ± SEM, Student’s *t*-tests

To examine whether EYA2(A510E) mutant increased the instability of EYA2 protein, we treated the HEK293T cell lines stably expressing EYA2 and EYA2(A510E) with cycloheximide (CHX), an inhibitor of new protein biosynthesis, to monitor the rate of protein degradation. Western blot analysis showed that EYA2 wild-type protein was relatively stable in HEK293T cells with a half-life greater than 7 h, however, the half-life was shortened to approximately 4 h in EYA2(A510E) expressing cells (Fig. 5A). We stably overexpressed Flag-tagged EYA2 and EYA2(A510E) mutant in Huh-7 and MHCC-97H cells and performed confocal immunofluorescence to detect the EYA2 and EYA2(A510E) expression at the same laser intensity. EYA2 protein was observed to distribute in the cytoplasm and nucleus, but the fluorescence intensity was markedly attenuated in EYA2(A510E)-transfected cells (Fig. 5B). The long side chain of E (glutamate) at position 510 in the mutant type was predicted to affect the spatial conformation and the correct folding of EYA2 protein compared to the A (alanine) in the wide-type structure (Fig. 5C).

EYA2(A510E) mutation led to the activation of the genes related to the unfolded protein response (UPR) such as ATF-6, IRE1α and PERK in HCC cell lines (Fig. 5D). The phosphorylation of PERK and IRE1α was markedly increased and the expression of ATF-6 and CHOP was upregulated in the mutant group compared with their phosphorylation and expression in the EYA2 wild-type group (Fig. 5D). Western blot analysis showed that both chloroquine and MG132 in the mutant group inhibited the degradation of EYA2 expression in a dose-dependent manner (Fig. 5E, F). Since MG132 is confirmed to induce endoplasmic reticulum stress by inhibiting the ubiquitin-proteasomal system [13]. EYA2(A510E) mutation promoted the activation of ATF-6, p-PERK, p-IRE1α and CHOP compared to EYA2 wild-type under the MG132 treatment (Additional file 3: Fig. S8). However the ubiquitination of EYA2(A510E) was not increased by the MG132 treatment (Fig. 5G), suggesting the degradation of EYA2(A510E) mutant was not related with ubiquitination. Immunofluorescent staining showed that the colocalization of Flag-tagged EYA2 with the lysosome marker Lysosomes-RFP was more intense in the EYA2(A510E) mutant group treated with chloroquine (Fig. 5H), and also the Flag-tagged EYA2 and the proteasome marker 20S were colocalized in the mutant group treated with MG132 (Fig. 5I). These results demonstrate that EYA2(A510E) mutation leads to protein degradation by inducing UPR via lysosome pathway and ubiquitination-independent proteasome pathway.

### EYA2 interacts with DACH1 to suppress HCC progression via SOCS3-mediated blockade of JAK/STAT signaling

**Fig. 6:**
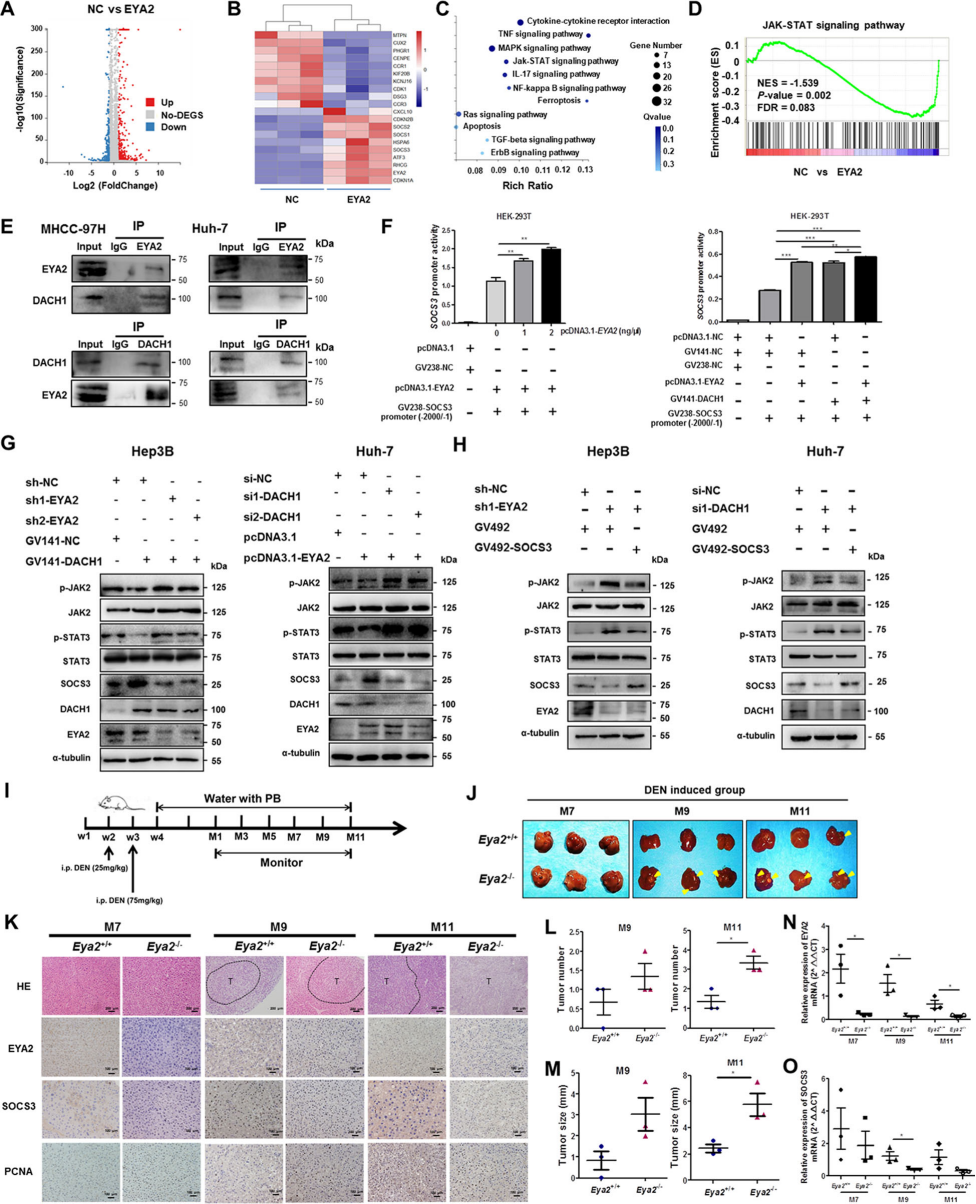
EYA2 interacts with DACH1 to suppress HCC progression via SOCS3-mediated blockade of JAK/STAT signaling. (A) Scatter plot showing the differentially expressed genes regulated by the overexpression of EYA2. (B) Heat map showing the representative differentially expressed genes modulated by the overexpression of EYA2. (C) KEGG pathway analysis showing the most enriched pathways of the differentially expressed genes. (D) GSEA showing the enrichment of JAK/STAT-related gene signatures in the EYA2 overexpression cells. (E) Co-immunoprecipitation of endogenous DACH1 with anti-EYA2 antibody (upper) and endogenous EYA2 with anti-DACH1 antibody (lower) in MHCC-97H and Huh-7 cells. (F) SOCS3 promoter constructs (− 2000/− 1) co-transfected with pcDNA3.1-EYA2 or/and GV141-DACH1 and the relative luciferase activity measured in HEK293T cells. (G) Expression of SOCS3, p-STAT3, p-JAK2, STAT3 and JAK2 in Hep3B and Huh-7 cells co-transfected with GV141-DACH1/sh-EYA2 or si-DACH1/pcDNA3.1-EYA2 detected by western blot. (H) Western blot analysis of expression of STAT3, p-STAT3, JAK2 and p-JAK2 in Hep3B and Huh-7 cells co-transfected with GV492-SOCS3/sh1-EYA2 or si1-DACH1/GV492-SOCS3. (I) Scheme of *Eya2*^−/−^ mice induced with DEN. (J) Images of the liver of *Eya2*^−/−^ and *Eya2*^+/+^ mice treated with DEN at 7, 9 and 11 months. (K) H&E staining and immunohistochemistry analysis of the expression of EYA2, SOCS3 and PCNA in liver tissues from mice treated with DEN at 7, 9 and 11 months. T indicates tumor nodule. Number of tumors (L) and tumor sizes (M) from mice treated with DEN at 9 and 11 months. qRT-PCR analysis of EYA2 (N) and SOCS3 (O) expression in liver tissues from mice treated with DEN at 7, 9 and 11 months (*n =* 3 for each group). *P < 0.05, **P < 0.01, ***P < 0.001. (F, L, M, N, O) mean ± SEM, Student’s *t*-tests

To investigate the mechanisms underlying EYA2 inhibition in HCC progression, we performed RNA sequencing to identify genes whose expression was changed by the stable overexpression of EYA2 using Huh-7-negative control and Huh-7-EYA2 cell lines. A total of 320 genes were down-regulated and 464 genes were up-regulated after the overexpression of EYA2 (Fig. 6A, B). A further KEGG pathway analysis showed that the differentially expressed genes were significantly related to several pathways including cytokine-cytokine receptor interaction (*P* = 3.97e-6), TNF signaling pathway (*P* = 2.43e-5), MAPK signaling pathway (*P* = 7.16e-5) and JAK/STAT signaling pathway (*P* = 1.59e-3) (Fig. 6C). Gene set enrichment analysis (GSEA) revealed that JAK/STAT signaling pathway was prominently inhibited in HCC with the overexpression of EYA2 (Fig. 6D). Previous studies had indicated an interaction of Eyes absent and Dachshund proteins by yeast two-hybrid analyses [14]. Here, we proved an interaction of EYA2 and DACH1 proteins by co-immunoprecipitation assay in MHCC-97H and Huh-7 cell lines (Fig. 6E). Notably, SOCS family members SOCS1, SOCS2 and SOCS3, negatively associated with activation of the JAK/STAT signaling pathway [15–17], were up-regulated after the overexpression of EYA2 (Fig. 6B). To validate the results observed by RNA-Seq on Huh-7 cell line, qRT-PCR was performed to detect eight representative genes and revealed similar profiles of gene expression (Additional file 3: Fig. S9A).

We also analyzed the DACH1, SOCS1, SOCS2 and SOCS3 mRNA expression using two GEO datasets, GSE22058 and GSE14520. The results showed that DACH1, SOCS2 and SOCS3 expressions were lower in HCC than in the adjacent tissues, but SOCS1 expression had no significant difference (Additional file 3: Fig. S9B). Furthermore, SOCS3 expression was positively correlated with the expression of EYA2 and DACH1. While SOCS2 was not significantly associated with EYA2, as well as no correlation between EYA2 and DACH1 (Additional file 3: Fig. S9C). A network involving crucial proteins EYA2, DACH1 and SOCS3 was shown in Additional file 3: Fig. S9D. Immunohistochemistry analysis of 60 cases of HCC tissues was performed to further verify the association between EYA2 and SOCS3 expression, which indicated that SOCS3 expression was significantly decreased in HCC compared with the paired adjacent tissues (Additional file 3: Fig. S10A,B). More importantly, EYA2 expression was positively associated with SOCS3 expression (r = 0.386, *P* = 0.002; Additional file 3: Fig. S10C). The Kaplan-Meier analysis indicated that HCC patients with high SOCS3 expression had a favorable prognosis (Additional file 3: Fig. S10D).

Based on the above analysis, we supposed that EYA2 combines with DACH1 to transcriptionally regulate the SOCS3 expression in inhibition of HCC. To test our hypothesis, we performed a promoter activity assay using a plasmid constructed by linking the SOCS3 promoter to a luciferase gene. The SOCS3 promoter showed a significant increase in the expression of luciferase associated to the increased EYA2 concentration, and the combination of EYA2 and DACH1 enhanced a higher transcriptional activation of SOCS3 than their alone (Fig. 6F; Additional file 3: Fig. S11A). The combined EYA2 and DACH1 regulated the expression of SOCS3 protein, which blocked the JAK/STAT signaling pathway (Fig. 6G; Additional file 3: Fig. S11B). Most importantly, we performed SOCS3 rescue experiments and found that p-JAK2 and p-STAT3 were up-regulated through EYA2 knockdown in Hep3B cells or DACH1 knockdown in Huh-7 cells, which could be retarded by SOCS3 overexpression (Fig. 6H). EYA2 expression in Hep3B cells and DACH1 in Huh-7 cells was not changed significantly after SOCS3 overexpression (Fig. 6H). Moreover, western blot analysis showed that SOCS3 expression was down-regulated in EYA2(A510E) mutant group compared with the expression in the EYA2 wild-type group (Additional file 3: Fig. S11C).

**Fig. 7:**
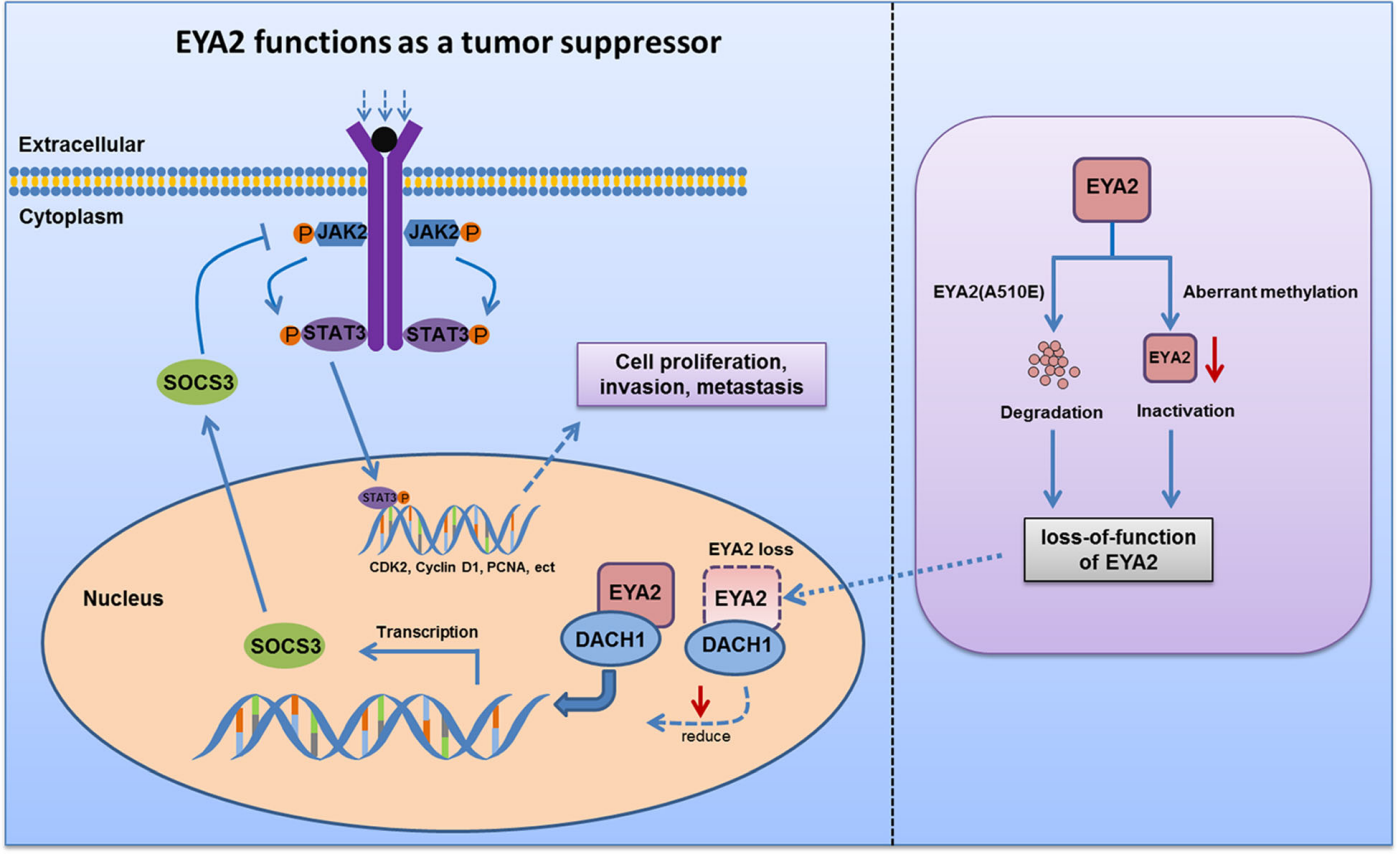
A proposed model for the tumor-suppressive activity of EYA2

To further explore the effect of EYA2 on inhibition of hepatocarcinogenesis, hepatocyte-specific EYA2 knockout (*Eya2*^−/−^) mice were established by the crossing of *Eya2*^1oxp/1oxp^ mice with the Alb-Cre mice (Additional file 3: Fig. S12A). The mice were then treated with diethylnitrosamine (DEN)/phenobarbital (PB) for induction of liver tumor (Fig. 6I). We sacrificed each cohort of induced mice at 7, 9, and 11 months, and liver tumors were found in 100% (3/3) of the *Eya2*^−/−^ mice at 9 months after DEN treatment, whereas no obviously macroscopic tumor was found in the *Eya2*^+/+^ group (Fig. 6J, K). Thus, the incidence of liver tumor was higher in *Eya2*^−/−^ mice compared with that of *Eya2*^+/+^ mice. Moreover, the number and size of liver tumors in *Eya2*^−/−^ mice at 11 months were significantly increased compared with those in the controls (Fig. 6L, M). However, the non-induced groups did not show any tumor development at 11 months (Additional file 3: Fig. S12B,C). Immunohistochemistry and qRT-PCR confirmed the depletion of EYA2 from the hepatocytes of the *Eya2*^−/−^ mice, and the expression of SOCS3 was reduced in *Eya2*^−/−^ mice than that in *Eya2*^+/+^ mice at each time point (Fig. 6K,N,O). The high expression of PCNA in *Eya2*^−/−^ mice indicated the active proliferation of tumor cells (Fig. 6K). To determine whether EYA2 is involved in STAT3 activation under the interleukin-6 (IL-6) stimulation, primary hepatocytes isolated from *Eya2*^−/−^ mice and control littermates were exposed to IL-6. Western blot assay revealed that STAT3 phosphorylation induced by IL-6 was significantly higher in EYA2-deficient hepatocytes than control littermates at each time point, indicating EYA2 plays a role in negative regulation of IL-6-induced STAT3 activation (Additional file 3: Fig. S13). Altogether, these results suggest that EYA2 acts as a tumor suppressor to prevent hepatocarcinogenesis and the malignant phenotype of HCC through the SOCS3-mediated blockade of JAK/STAT signaling, providing a molecular basis for the observed increased aggressiveness in EYA2 loss tumors (Fig. 7).

## Discussion

Hepatocarcinogenesis is a multi-stage and complex biological process driven by genetic alterations that inactivate tumor suppressor genes or activate oncogenes and abnormal epigenetic modifications [18]. Systematic analysis of tumor somatic mutations can be obtained by a combined high-throughput sequencing and bioinformatics analysis. To date, somatic alterations and mutation frequency in driver genes in HCC mainly include TERT (50%–60%), TP53 (12–48%), CTNNB1 (11–35%), AXIN1 (5–15%), ARID1A (4–17%), FGF19 (2–9%) and KEAP1 (2–8%), which are commonly involved in telomere maintenance, cell cycle, wnt/β-catenin, epigenetic modification, AKT/mTOR MAPK signaling pathway and oxidative stress regulation [19]. In the present work, we identified new somatic mutations of EYA2 at two sites (p.R255K and p.A510E) using the whole exome sequencing of HCC tissues. Interestingly, the mutation of the 510th amino acid of the EYA2 was also present in malignant melanoma (p.A510A) and gastric adenocarcinoma (p.A510V) in the COSMIC database. However, the significance of the mutation at 510th amino acid of EYA2 in cancers has remained elusive. It should be noted that although EYA2 mutations are rare in HCC and other cancers, they do occur. It will be interesting to determine whether there are somatic mutations or deletion at the EYA2 locus in HCC genomes that could result in functional inactivation of the tyrosine phosphatase or changes in binding capacity of transcriptional coactivator.

The tumor-suppressing role of EYA2 in HCC is unexpected because the previous experiments indicated a positive role of EYA2 in regulation of pro-metastatic characteristics of breast cells through the TGF-β pathway by working in concert with SIX1 protein [6]. Li et al. demonstrated that the EYA2 promoted cell proliferation through down-regulation of PTEN in lung cancer [7]. However, the Vincent’s group demonstrated that deletion of EYA2 promoted pancreatic adenocarcinomas progression through the disruption of TGF-β pathway [9]. In an attempt to assess the function of EYA2 on multiple cancer cells, we found that overexpression of EYA2 had a weak ability to affect tumor cell proliferation in HT-29, PANC-1 and MCF-7 cell lines, but sturdy in MHCC-97H, Huh-7 and A549 cell lines. This may be due to the different regulatory mechanisms of EYA2 and the complex tumor heterogeneity on tumor cell proliferation in different cancer cells. Of note, evidences obtained from both in vivo and in vitro experiments pointed towards EYA2 functions as a tumor suppressor in HCC. Notably, the growth rate and tumor weight of the subcutaneous transplanted human HCC-derived tumors with high EYA2 expression were significantly lower than those with low EYA2 expression in nude mice in vivo. Delivery of EYA2 gene had a therapeutic effect on inhibition of orthotopic liver tumor in nude mice. These findings suggest that EYA2 has organ or tissue specific functions, presumably depending on its subcellular localization and underlines the complexity of its double functions of a transcription coactivator and phosphatase during differentiation and cell cycle progression [20]. Specifically, the expression of EYA2 in adjacent tissues has a nuclear localization distribution, which may play a role in tumor-suppressive activities in the processes of intracellular signaling and transcription.

An aberrant methylation in the promoter region is an important cause of gene down-regulation in tumors. Vincent et al. reported that EYA2 is silenced in pancreatic cancer cell lines mainly because of promoter methylation [9]. In this report we used TCGA methylation data to analyze EYA2 methylation status of pan-cancer, in which we found EYA2 was hypermethylated in breast cancer than adjacent tissues. However, several studies reported that EYA2 promoted the progression of breast cancer [6, 21, 22]. This brings the complexity of cancer research, suggesting the methylation of EYA2 may not be the main cause modification of breast cancer progression to a great extent. Methylation modification also occurred in the 5’UTR and the first intron region of specific genes, and CpGs in these regions are methylated to prevent the correct binding of transcription factors, which can affect gene transcription [23, 24]. We here show that the methylation rate of EYA2 in the intron 1 region of the HCC tissues was significantly higher than that in the adjacent tissues, and this methylation rate was negatively correlated with the mRNA expression of EYA2 in the HCC tissues.

Rapidly growing evidence reinforces the notion that tumors are initiated by somatic driver mutations [25]. The point mutation in the gene exome region may cause conformational changes and protein instability, which can lead to protein degradation [26, 27]. Rosenberg et al. demonstrated that mutant PKCαD463H protein was less stable than PKCαWT, however, PKCαD463H enhanced proliferation and cell cycle progression of human astrocytes [26]. In this report we discovered that EYA2(A510E) mutation leads to protein degradation by UPR and then reduced the inhibitory function of wild type. Moreover, the ubiquitin-proteasome system and lysosome system are two major pathways leading to protein degradation [28]. The misfolded or incorrect structure proteins can also be degraded by non-ubiquitin-dependent degradation pathways under certain cellular stress conditions [29]. We confirmed that the degradation of EYA2(A510E) depended on the lysosomal degradation pathway and the non-ubiquitinated proteasome degradation pathway.

Indeed, a critical role of JAK/STAT in mediating tumorigenesis has been reported in several types of cancer [30]. SOCS3, a key negative regulator of the JAK/STAT pathway, is frequently down-regulated in HCC tissues and can inhibit malignant transformation of HCC cells [31]. Khan et al. reported that SOCS3 mRNA expression was significantly lower in HCC than adjacent tissues, yet SOCS3 mRNA expression lacked predictive potential [32]. However, high expression of SOCS3 protein in HCC patients had better prognosis than that for patients with SOCS3 low-expression [33, 34]. In our study, we also demonstrated that HCC patients with high SOCS3 protein expression had a better prognosis. SOCS3 interacts directly with JAK2 kinase, and inhibits JAK2 activity in an ATP-independent manner by partially blocking the kinase’s substrate binding groove with the kinase inhibitory region, thereby down-regulating JAK-dependent phosphorylation of STATs [35]. Wormald et al. demonstrated that SOCS3 was required for bi-phasic STAT3 tyrosine phosphorylation with IL-6 stimulation [36]. The SOCS3-deficient hepatocytes exhibited enhanced activation of multiple IL-6-dependent signaling pathways [37]. In this study, we demonstrated that EYA2 plays a role in negative regulation of IL-6-induced STAT3 activation. EYA2 has no recognized DNA binding activity, but possesses a transactivation domain [4]. DACH1 has been verified to be a transcriptional repressor that binds directly to specific DNA sites and simultaneously remains functionally validated and best-characterized EYA2-binding proteins [4]. Our present data indicated that EYA2 combines with DACH1 to transcriptionally elevate the SOCS3 expression, which in turn inhibits the activation of JAK/STAT pathway. Moreover, we generated *Eya2*^−/−^ mice and deletion of EYA2 aggravates tumor formation on HCC development in DEN-induced *Eya2*^−/−^ mice, supporting our theory that EYA2 plays an essential role in the retard of initiation and progression of HCC.

## Conclusions

The present work is the first reporting the role of EYA2 in suppressing the malignant phenotype of HCC, and the somatic mutation p.A510E of EYA2 that neutralized the tumor inhibitory effect of EYA2. Elucidating the roles of EYA2, acting as tumor suppressor will shed light on the molecular basis of HCC and may suggest therapeutic strategies for the malignant tumors.

## Supplementary Information

**Additional file 1: Supplementary materials and methods**.

**Additional file 2: Table S1**. Clinical characteristics of six patients with HCC. **Table S2**. Sequence analysis of six pairs of HCC primary tumors and matched adjacent tissues. **Table S3**. The 203 genes harboring non-silent SNVs or indels mutations. **Table S4**. Summary of the types and prevalence of the somatic mutations identified in six pairs of HCC primary tumors and matched adjacent tissues. **Table S5**. Clinical information of patients with HCC (*n* = 94). **Table S6**. Univariate and multivariate analysis of clinicopathological factors for overall survival rate (*n* = 94). **Table S7**. Comparison of HCC and adjacent non-tumors DNA methylation levels of each CpG site within 411 bp flanking region of EYA2 intron 1 (*n* = 30 pairs). **Table S8**. Primer sequences in the study.

**Additional file 3: Fig. S1.** The classification and number of somatic mutations identified by whole exome sequencing. **Fig. S2.** Somatic mutations identified in candidate gene GPR98, EYA2 and UBE2S, which were confirmed by IGV visualization from HCC and case-matched adjacent tissues. The bold numbers in the figures represent allelic fraction of the altered base. **Fig. S3.** The mRNA expression and function of EYA2 in pan-cancer. (A) mRNA expression of EYA2 in pan-cancer and their respective adjacent based on TCGA database. (B) mRNA expression of EYA2 in pan-cancer cells based on CCLE database. (C) Western blot analysis of the expression of EYA2 in four cancer cells transiently transfected with overexpression vectors. (D) The effect of pcDNA3.1-mediated overexpression of constructs encoding EYA2 wild-type on cell proliferation of cancer cells in vitro. (E) The effect of EYA2 wild-type on cell invasion of cancer cells in vitro. ACC, adrenocortical carcinoma; BLCA, bladder urothelial carcinoma; BRCA, breast invasive carcinoma; CESC, cervical squamous cell carcinoma and endocervical adenocarcinoma; CHOL, cholangiocarcinoma; COAD, colon adenocarcinoma; DLBC, lymphoid neoplasm diffuse large B-cell lymphoma; ESCA, esophageal carcinoma; GBM, glioblastoma multiforme; HNSC, head and neck squamous cell carcinoma; KICH, kidney chromophobe; KIRC, kidney renal clear cell carcinoma; KIRP, kidney renal papillary cell carcinoma; LAML, acute myeloid leukemia; LGG, brain lower grade glioma; LIHC, liver hepatocellular carcinoma; LUAD, lung adenocarcinoma; LUSC, lung squamous cell carcinoma; MESO, mesothelioma; OV, ovarian serous cystadenocarcinoma; PAAD, pancreatic adenocarcinoma; PCPG, pheochromocytoma and paraganglioma; PRAD, prostate adenocarcinoma; READ, rectum adenocarcinoma; SARC, sarcoma; SKCM, skin cutaneous melanoma; STAD, stomach adenocarcinoma; TGCT, testicular germ cell tumors; THCA, thyroid carcinoma; THYM, thymoma; UCEC, uterine corpus endometrial carcinoma; UCS, uterine carcinosarcoma; UVM, uveal melanoma. *P < 0.05, **P < 0.01, ***P < 0.001. (A, D, E) mean ± SEM, Student’s *t*-tests. **Fig. S4.** EYA2 inhibits the malignant phenotypes of HCC cells in vitro. (A) Western blot analysis of the expression of EYA2 in five liver cancer cells. (B) Expression of EYA2 in MHCC-97H cells or Hep3B transfected or infected with si-EYA2 or sh-EYA2 detected by qRT-PCR analysis. (C) Western blot analysis of the expression of EYA2 in MHCC-97H or Hep3B cells stably transfected with shRNA or EYA2 lentivirus and EYA2(A510E) mutant lentivirus. The effect of shRNA-mediated knockdown of EYA2 and lentivirus-mediated EYA2 and EYA2(A510E) overexpression on cell proliferation (D) and cell invasion (E) HCC cells in vitro. The flow cytometry analysis of the effect of EYA2 on cell cycle progression (F) and apoptosis (G) of HCC cells. (H) Western blot analysis of P27, BAX, cyclin D1 and CDK2 expression in HCC cells stably transfected with sh-EYA2 or the overexpression vectors. *P < 0.05, **P < 0.01, ***P < 0.001. (B, D, E–G) mean ± SEM, Student’s *t*-tests. **Fig. S5.** Expression of EYA2 in tumor tissues detected by western blot analysis. *P < 0.05, mean ± SEM, Student’s *t*-tests. **Fig. S6.** Association of the mRNA expression of EYA2 with its methylation level and copy number analyses in different types of tumors. (A) Potential CpG islands of EYA2 were predicted from 5000 bp upstream to 1000 bp downstream of the transcription start site. (B) The methylation level of EYA2 in pan-cancer and their respective adjacent tissues based on TCGA database. (C) Correlation between EYA2 methylation and the mRNA expression in pan-cancer. (D) Copy number distribution of the known genes and EYAs family gene based on TCGA_LIHC (The blue line in the inner circle represented the deletion and the black line in the inner circle represented the gain). (E) Correlation between EYA2 copy number and the mRNA expression. BLCA, bladder urothelial carcinoma; BRCA, breast invasive carcinoma; COAD, colon adenocarcinoma; ESCA, esophageal carcinoma; HNSC, head and neck squamous cell carcinoma; KIRC, kidney renal clear cell carcinoma; KIRP, kidney renal papillary cell carcinoma; LUAD, lung adenocarcinoma; LUSC, lung squamous cell carcinoma; PAAD, pancreatic adenocarcinoma; PRAD, prostate adenocarcinoma; THCA, thyroid carcinoma; UCEC, uterine corpus endometrial carcinoma. *P < 0.05, **P < 0.01, ***P < 0.001. (B) mean ± SEM, Student’s *t*-tests; (C) Pearson’s correlation test; (E) Kruskal-Wallis test. **Fig. S7.** EYA2(A510E) mutation reduces EYA2 protein level. qRT-PCR (A) and western blot (B) analysis of EYA2 expression in HEK293T and Huh-7 transiently transfected with overexpression and mutant vectors. qRT-PCR (C) and western blot (D) analysis of EYA2 expression in HEK293T and two HCC cell lines MHCC-97H, Huh-7 stably infected with lentivirus-EYA2 and lentivirus-EYA2(A510E). *P < 0.05, **P < 0.01, ***P < 0.001. (A, C) mean ± SEM, Student’s *t*-tests. **Fig. S8.** Western blot analysis of unfold protein response-related protein expression in MHCC-97 cells treated with MG132. **Fig. S9.** Association of EYA2 with DACH1, SOCS1, SOCS2 and SOCS3 expression in human HCC tissues. (A) qRT-PCR verification of eight representative genes in Huh-7 cells with EYA2 overexpression. Expression of SOCS1, SOCS2, SOCS3 and DACH1 in HCC and adjacent tissues (B) and correlation of EYA2 with SOCS2, SOCS3 and DACH1 in human HCC tissues (C) based on GSE22058 and GSE14520 database. (D) The network interaction diagram of crucial genes EYA2, DACH1 and SOCS3. **Fig. S10.** Expression and prognostic significance of SOCS3 in HCC tissues. (A) A representative immunohistochemical staining of SOCS3 in HCC and case-matched adjacent tissues. (B) Immunohistochemistry scores associated to the expression of SOCS3 in HCC and paired adjacent tissues (*n* = 60 pairs). (C) Correlation analysis between EYA2 and SOCS3 protein expression (*n =* 60 cases). (D) Kaplan-Meier analysis of overall survival of HCC patients with high or low SOCS3 expression (*n =* 60 cases). ***P < 0.001. (B) mean ± SEM, Student’s *t*-tests; (C) Spearman’s correlation test. (D) Kaplan-Meier analysis. **Fig. S11.** The combination of EYA2 and DACH1 regulates the SOCS3-mediated blockade of JAK/STAT signaling. (A) SOCS3 promoter constructs (− 2000/− 1) co-transfected with pcDNA3.1-EYA2 or/and GV141-DACH1 and the relative luciferase activity measured in MHCC-97H cell line. (B) Western blot analysis of expression of SOCS3, p-STAT3, p-JAK2, STAT3 and JAK2 in MHCC-97H cells co-transfected with si-DACH1/pcDNA3.1-EYA2. (C) Western blot analysis of expression of SOCS3 in MHCC-97H and Huh-7 cells infected with lentivirus-EYA2 and lentivirus-EYA2(A510E) mutant. *P < 0.05. (A, B) mean ± SEM, Student’s *t*-tests. **Fig. S12.** Detection of *Eya2*^−/−^ and *Eya2*^+/+^ mice of non-induced group at 11 months. (A) DNA electrophoretic map of genotype identification of *Eya2*^−/−^ mice. Electrophoresis of the PCR products of the Alb-Cre gene (left) and EYA2 (right). *Eya2*^+/+^ represented wild-type mouse with a band size of 278 bp and *Eya2*^−/−^ represented hepatocyte-specific EYA2 knockout mouse with a band size of 358 bp. (B) Images of the liver from non-induced *Eya2*^−/−^ and *Eya2*^+/+^ mice. (C) H&E staining and immunohistochemistry analysis of the expression of EYA2, SOCS3 and PCNA in liver tissues from the non-induced mice. **Fig. S13.** The level of STAT3 phosphorylation in response to IL-6 in *Eya2*^−/−^ hepatocytes. STAT3 phosphorylation induced by IL-6 was significantly higher in EYA2-deficient hepatocytes than control littermates at each point of time.

## Data Availability

The accession numbers for the whole exome sequencing and RNA-seq data reported in this paper are Sequence Read Archive (SRA): PRJNA675890 and PRJNA675419. The datasets and materials used or analyzed during the current study are available from the corresponding author on reasonable request.

## Abbreviations

BCLC: Barcelona Clinic Liver Cancer
CCLE: Cancer Cell Line Encyclopedia
CHX: Cycloheximide
DEN: Diethylnitrosamine
EYA2: Eyes absent homolog 2
GSEA: Gene set enrichment analysis
HCC: Hepatocellular carcinoma
H&E: Hematoxilin-eosin
IGV: Integrated Genomics Viewer
PB: Phenobarbital
PBS: Phosphate-buffered saline
TCGA: The Cancer Genome Atlas
UPR: Unfolded protein response
5-azaC: 5-aza-2′-deoxycytidine
