# Supplementary material for: EYA2 suppresses the progression of hepatocellular carcinoma via SOCS3-mediated blockade of JAK/STAT signaling
Source: Mol Cancer. 2021 May 27;20:79. doi: 10.1186/s12943-021-01377-9 (PMC8157759; doi:10.1186/s12943-021-01377-9)
Supplement: Supplementary file 1 — Additional file 1: Supplementary materials and methods. [file 12943_2021_1377_MOESM1_ESM.docx]

**Supplementary Information**

**Supplementary materials and methods**

**Whole-exome sequencing**

Genomic DNA libraries were prepared using protocols recommended by Illumina. The detailed information about sequencing was described in our previous study [1].

**Identification and validation of candidate somatic mutations**

The pipeline applied for the identification of somatic mutations based on the Illumina sequencing data was described in our previous study [1]. Candidate mutations were visualized using the Integrated Genomics Viewer (IGV) [2]. PCR amplification and Sanger were performed following standard protocols using the primers listed in Additional file 2: Table S8 to validate the candidate somatic mutations after exome sequencing. The function effects of the candidate mutations were predicted by Polymorphism Phenotyping v2 (Polyphen-2, http://genetics.bwh.harvard.edu/pph2/index.shtml) and Scale-invariant feature transform (SIFT, http://sift.jcvi.org/).

**Public data on liver cancer**

Publicly available data were generated from The Cancer Genome Atlas (TCGA) (https://portal.gdc.cancer.gov/) and downloaded from UCSC Xena (http://xena.ucsc.edu/), including expression data and methylation data of pan-cancer and copy number data of liver cancer. The mRNA expression values of five genes EYA2, DACH1, SOCS1, SOCS2 and SOCS3 for TCGA_LIHC were subjected to log2 (FPKM + 1) transformation. The methylation of EYA2 CpG islands data in TCGA_LIHC was downloaded from MethHC database (http://methhc.mbc.nctu.edu.tw/). The mRNA expression data of EYA2 in liver cancer were downloaded from Gene Expression Omnibus (GEO, http://www.ncbi.nlm.nih.gov/geo/) under the accession number GSE22058 and GSE14520. The mRNA expression and genetic alteration data of EYA2 were downloaded from International Cancer Genome Consortium (ICGC) (https://icgc.org/) and Catalogue of Somatic Mutations in Cancer (COSMIC, v91) (https://cancer.sanger.ac.uk/cosmic).

**RNA sequencing and gene set enrichment analysis**

The RNA-seq analysis was performed by BGI (Shenzhen, China). Genes were considered differentially expressed when log2 (fold change) was greater than 1 or less than -1 with a false discovery rate (FDR, q) < 0.001 [3]. Kyoto Encyclopedia of Genes and Genomes pathway analysis was done using the phyper function in R (version 3.6.0) software.

Gene Set Enrichment Analysis (GSEA) (version 3.0, the broad institute of MIT and Harvard, http://software.broadinstitute.org/gsea/downloads.jsp) was performed to investigate the biological characteristics of HCC with the overexpression of EYA2. The specific parameters of GSEA were described in our previous study [4].

**Computational modeling of wild-type and mutant EYA2 protein**

The available protein crystal structure of the human EYA2 ED domain (PDB: 4EGC) was used as the template [5]. The structural images were produced using PyMOL software (version 2.3.2_0) (DeLano Scientific LLC, CA, USA).

**Antibodies and reagents**

The EYA2 (11314-1-AP), α-tubulin (11224-1-AP), ATF-6 (24169-1-AP), CHOP (15204-1-AP), DACH1 (10914-1-AP) and SOCS3 (14025-1-AP) were purchased from Proteintech (Wuhan, China). Flag-Tag (8146S), Stat3 (9139S), Phospho-STAT3 (9145S), Phospho-JAK2 (66245S), JAK2 (3230T), PERK (5683T), Phospho-PERK (3179S) and IRE1α (3294T) were purchased from Cell Signaling Technology (MA, USA). Phospho-IRE1α (ab48187) and Anti-Proteasome 20S (ab3329) were obtained from Abcam (Cambridge, UK). Ubiquitin (sc-271289) was purchased from Santa Cruz Biotechnology (TX, USA). CellLight^TM^ Lysosomes-RFP (C10597, BacMam 2.0) was purchased from ThermoFisher Scientific (MA, USA). MG132 (proteasome inhibitor), cycloheximide (100 μg/ml), 5-aza-2’-deoxycytidine (5 μM), Dimethyl sulfoxide (D2650) and chloroquine (autophagy inhibitor) were from Sigma (MO, USA). The 5-aza-2’-deoxycytidine was dissolved in dimethyl sulfoxide in our study. Recombinant mouse interleukin-6 (IL-6) (HY-P7063) was purchased from MCE company (NJ, USA).

**Cell lines**

The human HCC cell lines, HepG2, SK-HEP-1 and Hep3B were obtained from the American Type Culture Collection (ATCC) (VA, USA). Huh-7 was obtained from the Japanese Collection of Research Bioresources (JCRB, Osaka, Japan) and the human HCC cell line, MHCC-97H, was purchased from the Cell Bank of the Chinese Academy of Sciences (Shanghai, China). HEK293T, HT-29, PANC-1, MCF-7 and A549 were from the ATCC. All cell lines used in our study was identified via STR profiles by Genetic Testing Biotechnology Co. (Suzhou, China). All cell lines were culture in RPMI 1640 medium containing 1% penicillin and streptomycin and 10% fetal bovine serum in 5% CO_2_ at 37°C.

**Transfection**

EYA2 siRNAs were designed and synthesized by GenePharma Co, Ltd. (Shanghai, China), and sequences for siRNAs were shown in Additional file 2: Table S8. The plasmids pcDNA3.1-EYA2, pcDNA3.1-R255K and pcDNA3.1-A510E were constructed by our laboratory and verified by sequencing (BGI, China). The three plasmids containing GV492-SOCS3, GV238-SOCS3 promoter and GV141-DACH1 were purchased from Genechem Co., Ltd. (Shanghai, China). Lentivirus carrying EYA2, EYA2(A510E) and sh-EYA2 were purchased from GeneCopoeia for constructing stable cell lines (Guangzhou, China). Cells were transiently transfected with plasmids or siRNA using Lipofectamine 2000 (Invitrogen, CA, USA) according to the manufacturer’s instructions.

**Cell proliferation, clone formation, migration, matrigel invasion assays *in vitro***

Huh-7, MHCC-97H and Hep3B cells with EYA2 overexpression or silencing were seeded into 96-well plates (5×10^3^ cells per well). Cell proliferation was measured every 24 hours by CCK-8 assays according to the manufacturer’s instructions. Invasion assays *in vitro* was performed using transwell chambers coated with matrigel. HCC cells treated with plasmids or lentivirus for 6 to 12 hours were seeded in serum-free medium in the upper chamber. Medium containing 10% fetal bovine serum was added into the lower chamber. After incubation for 24 hours or 48 hour at 37°C, HCC cells on the upper membrane were removed with cotton. The transwell chambers were stained with 0.1% crystal violet. Five fields of cells on the lower membrane were randomly photographed. Cells passing through the chambers were also stained with 0.1% crystal violet followed by eluting with acetic acid decolorization, which was measured the OD value at a wavelength of 570 nm on a microplate reader. Wound healing assay was used to evaluate cell migration. The cells confluent over 90% in a six-well plate replaced with serum-free medium for created wound with a 100 μl pipet tip. After 24 hours, the closure speed and spacing of this slit was measured by an inverted microscope (Nikon, Tokyo, Japan) and the wound closure analysis was performed through ImageJ software according to the formula: cell healing rate = ((measurement at 0 h – measurement at 24 h) / measurement at 0 h) ×100. Plate clone formation assay was applied to examine the cells dependence and proliferation. The 1×10^3^ cells were seeded on a 9 cm-dish for 7 days and then was stained with 0.1% crystal violet. The clones of five fields were counted at random under a microscope.

**Flow cytometry analysis**

Cells (1×10^5^) were transfected with pcDNA3.1-EYA2 for 48 hours, and then harvested and washed with phosphate-buffered saline (PBS) before exposure to staining buffer. The annexin V and PI stainings were performed using FITC/Annexin V-PI apoptosis detection kit (640914, BioLegend) according to the manufacturer’s instructions. For cell cycle analysis, harvested cells were fixed with 75% ethanol, washed twice with cold PBS and centrifuged to discard the supernatant, and then the cells were treated with a cell cycle kit (KGA512, KeyGEN Biotech), according to the manufacturer’s instructions. The data were analyzed using FACSCalibur instrument and cell quest software (BD Biosciences, NJ, USA).

**Quantitative real-time PCR**

Total RNA was extracted using the total RNA kit II (Omega, CA, USA) and was reverse transcribed into complementary DNA by PrimeScript^TM^ RT reagent kit (TaKaRa Biotechnology, Otsu, Japan). Single stranded complementary DNA was amplified by quantitative real-time PCR (qRT-PCR) using the SYBR Premix ExTaq kit (TaKaRa Biotechnology) in the Mx3005P Real-Time PCR system (Agilent Technologies, Germany). The primer sequences were listed in Additional file 2: Table S8.

**Immunofluorescence**

The cells in the dish were fixed with 4% paraformaldehyde and permeabilized with 0.2% Triton X-100 for 10 minutes. After PBS washing with three times, the cells were blocked with 10% goat serum for 30 minutes and incubated with primary antibodies at 4°C overnight. Cells were stained for 2 hours with Alexa 555-conjugated donkey anti-rabbit IgG (A31572), Alexa 555-conjugated donkey anti-mouse IgG (A31570), Alexa 488-conjugated donkey anti-rabbit IgG (A21206) or Alexa 488-conjugated donkey anti-mouse IgG (A21202) from Invitrogen (Carlsbad, USA). Cell nuclei was stained by the 4',6-diamidino-2-phenylindole (DAPI) (Thermo Scientific, MA, USA). The fluorescent was photographed under a confocal laser scanning microscope using Nikon NIS-Elements software (Nikon, Tokyo, Japan).

**Co-immunoprecipitation and western blot analysis**

The interaction of EYA2 and DACH1 in MHCC-97H and Huh-7 cells were detected using Co-immunoprecipitation kit (Pierce, 26149) according to the manufacturer’s instructions. Western blot analysis was performed according to a standard protocol, and the proteins were electrophoresed by SDS-PAGE and transferred to PVDF membranes (Millipore). Membranes were blocked in 5% skimmed milk and incubated with primary antibodies at 4°C overnight, and then labeled with anti-mouse IgG (Pierce, IL, USA) or anti-rabbit IgG (Pierce) secondary antibody at room temperature for 1 hour. The protein signal was visualized using a Western-Light chemiluminescent detection system (Image Station 4000 MM Pro, MA, USA).

**DNA extraction and methylation analyses**

Genomic DNA was extracted from 30 paired of HCC and the adjacent tissues using the genomic DNA rapid extraction kit (TIANGEN BIOTECH CO, LTD, Beijing, China). The quality and amount of genomic DNA were evaluated through gel electrophoresis and the NanoDrop 2000c (Thermo Scientific, MA, USA), respectively. The DNA methylation sequencing was performed by BGI (Shenzhen, China). The Sequenom MassARRAY platform (Agena, Inc, CA, USA) was performed for quantitative analysis of EYA2 methylation. The methylation was analyzed using EpiTYPER^TM^ software (Agena, Inc, CA, USA) to produce the original CpGs data and dot graph.

**Luciferase reporter assay**

The cells plated in a 24-well plate were co-transfected with 450 ng of the expression plasmids, 150 ng of the promoter reporter plasmids and 20 ng of pRL-TK (Promega, WI, USA) in HEK293T and MHCC-97H cells using Lipofectamine 2000 (Invitrogen, CA, USA). The luciferase reporter assay (Promega, WI, USA) was performed to detect luciferase activity according to the manufacturer’s protocol.

**Histopathology**

All liver tissue samples were embedded in paraffin after being fixed in 10% formalin for 24 hours, and then 3 μm slices were cut. Hematoxilin-eosin (H&E) staining was performed to evaluate the morphological changes.

**Immunohistochemistry**

The formalin-fixed and paraffin-embedded tissues were deparaffinized using xylene and ethyl alcohol, and then antigen retrieval was performed with citrate buffer (pH6.0). Sections were blocked with 0.3% H_2_O_2_ and normal goat serum, and then incubated with primary antibodies at 4°C overnight in a moist chamber. Subsequently, the sections were stained with a streptavidin-peroxidase staining kit (Zhongshan Jinqiao Co., Beijing, China) and the target proteins were imaged with 3,3'-diaminobenzidine (Zhongshan Jinqiao Co., Beijing, China).

All immunohistochemistry staining was independently assessed by two experienced pathologists. The staining intensity was scored as 0 (no staining), 1 (weakly positive), 2 (moderately positive), 3 (strongly positive). The area of positive staining was graded as 0 (0%–25%), 1 (26%–50%), 2 (51%–75%) and 3 (76%–100%). According to the immunohistochemistry score (staining intensity × positive percentage), the expression of EYA2 and SOCS3 were categorized as low expression (immunohistochemistry score 0–3) and high expression (immunohistochemistry score 4–9).

**Animal studies**

All mice were housed in a virus-free facility and placed in a temperature and light (12 hours light/dark cycle) controlled animal facility.

To detect the inhibition effect of EYA2 on the tumorigenicity of HCC cells, 1×10^6^ MHCC-97H or Hep3B cells with stably knockdown or stably overexpressing EYA2 were subcutaneously injected into the flanks of 6-week-old male nude mice, the nude mice were purchased from Chongqing Tengxin Biotechnology Co. LTD (Chongqing, China). When the tumors were visible to the naked eye and no more than 20 mm, the mice would be sacrificed. Tumor volume was calculated as follows: V (volume) = (length × width^2^)/2.

To evaluate the therapeutic effect of EYA2 *in vivo*, the tumors from the subcutaneous xenograft model were cut into 1 mm^3^ pieces and implanted into liver capsule of nude mice (male, 6-week-old) to simulate primary HCC. After two weeks, the 1×10^7^ titer unit (TU) and 2×10^7^ TU lentivirus carrying the EYA2 (Genechem Co., Ltd) were then injected via the tail vein twice in a week. The mice were sacrificed after 10 days of lentivirus injection.

*Eya2*^loxP/loxP^ mice with a loxP-flanked EYA2 allele on a C57BL/6 background were generated by CRISPR/Cas9 (Biocytogen, Beijing, China). The Albumin-Cre (Alb-Cre) mice were purchased from Shanghai Model Organisms Center, Inc (China). Alb-Cre mice were crossed with *Eya2*^loxP/loxP^ mice to produce Alb-Cre^+^/*Eya2*^loxP/loxP^ mice (*Eya2*^-/-^) and Alb-Cre^−^/*Eya2*^loxP/loxP^ (*Eya2*^+/+^) mice. Male *Eya2*^-/-^ and *Eya2*^+/+^ (three mice in each group) were used for the study. Mice were genotyped by PCR using the genomic DNA of mouse tail and the sequences of the genotyping primers are listed in Additional file 2: Table S8.

To induce HCC, hepatocyte-specific EYA2 knockout mice (*Eya2*^-/-^) and *Eya2*^+/+^ littermates were intraperitoneally injected with two doses (25 mg/kg and 75 mg/kg) of diethylnitrosamine (DEN, Sigma, MO, USA) on postnatal day 14 and day 21, respectively. Subsequently, the mice were fed water containing phenobarbital (PB) solvent, which is an enhancer of tumor formation [6,7]. Liver tissues were collected at 7, 9 and 11 months after birth for histopathologically H&E staining.

**Mouse primary hepatocyte isolation and culture**

Primary hepatocytes were isolated from EYA2 knock out (*Eya2*^-/-^) mice and *Eya2*^+/+^ littermates. The process used for mouse primary hepatocyte isolation was mainly based on a two-step collagenase perfusion method. In brief, hepatocytes were isolated from anesthetized 8-week-old male mice by non-recirculating perfusion of collagenase IV (C5138, Sigma-Aldrich, MO, USA) through the portal vein. The isolated cells were then filtered through a nylon filter and centrifuged with Percoll solution (P8370, Solarbio, Beijing, China). Finally, the viable cells at the bottom of the Percoll gradient were collected as primary hepatocytes and cultured in William’s E medium (Gibco, A1217601). After the cells completely adhered to the wall, 20 ng/ml of IL-6 was added to media.

**References:**

1. Liu ZK, Shang YK**,** Chen ZN, Bian H. A three-caller pipeline for variant analysis of cancer whole-exome sequencing data. Mol Med Rep. 2017;15:2489-2494
2. Robinson JT, Thorvaldsdóttir H, Winckler W, Guttman M, Lander ES, Getz G, et al. Integrative genomics viewer. Nat Biotechnol. 2011;29:24-26.
3. Wang L, Feng Z, Wang X, Wang X, Zhang X. DEGseq: an R package for identifying differentially expressed genes from RNA-seq data. Bioinformatics. 2010;26:136-138.
4. Liu ZK, Zhang RY**,** Yong YL, Zhang ZY, Li C, Chen ZN, et al. Identification of crucial genes based on expression profiles of hepatocellular carcinomas by bioinformatics analysis. Peerj. 2019;7:e7436.
5. Patrick AN, Cabrera JH, Smith AL, Chen XS, Ford HL, Zhao R. Structure-function analyses of the human SIX1-EYA2 complex reveal insights into metastasis and BOR syndrome. Nat Struct Mol Biol. 2013;20:447-453.
6. Tetri S, Ruhanen M, Viitala P, Pelkonen O, Pasanen M, Raunio H. Lack of association between CYP2A5 induction and apoptosis in mouse primary hepatocytes. Biochem Pharmacol. 2002;63:429-435.
7. Kinoshita A, Wanibuchi H, Imaoka S, Ogawa M, Masuda C, Morimura K, et al. Formation of 8-hydroxydeoxyguanosine and cell-cycle arrest in the rat liver via generation of oxidative stress by phenobarbital: association with expression profiles of p21(WAF1/Cip1), cyclin D1 and Ogg1. Carcinogenesis. 2002;23:341-349.

**Supplementary Figures Legends**

**Figure S1.** The classification and number of somatic mutations identified by whole exome sequencing.

**Figure S2.** Somatic mutations identified in candidate gene GPR98, EYA2 and UBE2S, which were confirmed by IGV visualization from HCC and case-matched adjacent tissues. The bold numbers in the figures represent allelic fraction of the altered base.

**Figure S3.** The mRNA expression and function of EYA2 in pan-cancer. (A) mRNA expression of EYA2 in pan-cancer and their respective adjacent based on TCGA database. (B) mRNA expression of EYA2 in pan-cancer cells based on CCLE database. (C) Western blot analysis of the expression of EYA2 in four cancer cells transiently transfected with overexpression vectors. (D) The effect of pcDNA3.1-mediated overexpression of constructs encoding EYA2 wild-type on cell proliferation of cancer cells *in vitro*. (E) The effect of EYA2 wild-type on cell invasion of cancer cells *in vitro*. ACC, adrenocortical carcinoma; BLCA, bladder urothelial carcinoma; BRCA, breast invasive carcinoma; CESC, cervical squamous cell carcinoma and endocervical adenocarcinoma; CHOL, cholangiocarcinoma; COAD, colon adenocarcinoma; DLBC, lymphoid neoplasm diffuse large B-cell lymphoma; ESCA, esophageal carcinoma; GBM, glioblastoma multiforme; HNSC, head and neck squamous cell carcinoma; KICH, kidney chromophobe; KIRC, kidney renal clear cell carcinoma; KIRP, kidney renal papillary cell carcinoma; LAML, acute myeloid leukemia; LGG, brain lower grade glioma; LIHC, liver hepatocellular carcinoma; LUAD, lung adenocarcinoma; LUSC, lung squamous cell carcinoma; MESO, mesothelioma; OV, ovarian serous cystadenocarcinoma; PAAD, pancreatic adenocarcinoma; PCPG, pheochromocytoma and paraganglioma; PRAD, prostate adenocarcinoma; READ, rectum adenocarcinoma; SARC, sarcoma; SKCM, skin cutaneous melanoma; STAD, stomach adenocarcinoma; TGCT, testicular germ cell tumors; THCA, thyroid carcinoma; THYM, thymoma; UCEC, uterine corpus endometrial carcinoma; UCS, uterine carcinosarcoma; UVM, uveal melanoma. **P* < 0.05, ***P* < 0.01, ****P* < 0.001. (A, D, E) mean ± SEM, Student’s *t*-tests.

**Figure S4.** EYA2 inhibits the malignant phenotypes of HCC cells *in vitro*. (A) Western blot analysis of the expression of EYA2 in five liver cancer cells. (B) Expression of EYA2 in MHCC-97H cells or Hep3B transfected or infected with si-EYA2 or sh-EYA2 detected by qRT-PCR analysis. (C) Western blot analysis of the expression of EYA2 in MHCC-97H or Hep3B cells stably transfected with shRNA or EYA2 lentivirus and EYA2(A510E) mutant lentivirus. The effect of shRNA-mediated knockdown of EYA2 and lentivirus-mediated EYA2 and EYA2(A510E) overexpression on cell proliferation (D) and cell invasion (E) HCC cells *in vitro*. The flow cytometry analysis of the effect of EYA2 on cell cycle progression (F) and apoptosis (G) of HCC cells. (H) Western blot analysis of P27, BAX, cyclin D1 and CDK2 expression in HCC cells stably transfected with sh-EYA2 or the overexpression vectors. **P* < 0.05, ***P* < 0.01, ****P* < 0.001. (B, D, E–G) mean ± SEM, Student’s *t*-tests.

**Figure S5.** Expression of EYA2 in tumor tissues detected by western blot analysis. **P* < 0.05, mean ± SEM, Student’s *t*-tests.

**Figure S6.** Association of the mRNA expression of EYA2 with its methylation level and copy number analyses in different types of tumors. (A) Potential CpG islands of EYA2 were predicted from 5000 bp upstream to 1000 bp downstream of the transcription start site. (B) The methylation level of EYA2 in pan-cancer and their respective adjacent tissues based on TCGA database. (C) Correlation between EYA2 methylation and the mRNA expression in pan-cancer. (D) Copy number distribution of the known genes and EYAs family gene based on TCGA_LIHC (The blue line in the inner circle represented the deletion and the black line in the inner circle represented the gain). (E) Correlation between EYA2 copy number and the mRNA expression. BLCA, bladder urothelial carcinoma; BRCA, breast invasive carcinoma; COAD, colon adenocarcinoma; ESCA, esophageal carcinoma; HNSC, head and neck squamous cell carcinoma; KIRC, kidney renal clear cell carcinoma; KIRP, kidney renal papillary cell carcinoma; LUAD, lung adenocarcinoma; LUSC, lung squamous cell carcinoma; PAAD, pancreatic adenocarcinoma; PRAD, prostate adenocarcinoma; THCA, thyroid carcinoma; UCEC, uterine corpus endometrial carcinoma. **P* < 0.05, ***P* < 0.01, ****P* < 0.001. (B) mean ± SEM, Student’s *t*-tests; (C) Pearson’s correlation test; (E) Kruskal-Wallis test.

**Figure S7.** EYA2(A510E) mutation reduces EYA2 protein level. qRT-PCR (A) and western blot (B) analysis of EYA2 expression in HEK293T and Huh-7 transiently transfected with overexpression and mutant vectors. qRT-PCR (C) and western blot (D) analysis of EYA2 expression in HEK293T and two HCC cell lines MHCC-97H, Huh-7 stably infected with lentivirus-EYA2 and lentivirus-EYA2(A510E). **P* < 0.05, ***P* < 0.01, ****P* < 0.001. (A, C) mean ± SEM, Student’s *t*-tests.

**Figure S8.** Western blot analysis of unfold protein response-related protein expression in MHCC-97 cells treated with MG132.

**Figure S9.** Association of EYA2 with DACH1, SOCS1, SOCS2 and SOCS3 expression in human HCC tissues. (A) qRT-PCR verification of eight representative genes in Huh-7 cells with EYA2 overexpression. Expression of SOCS1, SOCS2, SOCS3 and DACH1 in HCC and adjacent tissues (B) and correlation of EYA2 with SOCS2, SOCS3 and DACH1 in human HCC tissues (C) based on GSE22058 and GSE14520 database. (D) The network interaction diagram of crucial genes EYA2, DACH1 and SOCS3.

**Figure S10.** Expression and prognostic significance of SOCS3 in HCC tissues. (A) A representative immunohistochemical staining of SOCS3 in HCC and case-matched adjacent tissues. (B) Immunohistochemistry scores associated to the expression of SOCS3 in HCC and paired adjacent tissues (n = 60 pairs). (C) Correlation analysis between EYA2 and SOCS3 protein expression (n = 60 cases). (D) Kaplan-Meier analysis of overall survival of HCC patients with high or low SOCS3 expression (n = 60 cases). ****P* < 0.001. (B) mean ± SEM, Student’s *t*-tests; (C) Spearman's correlation test. (D) Kaplan-Meier analysis.

**Figure S11.** The combination of EYA2 and DACH1 regulates the SOCS3-mediated blockade of JAK/STAT signaling. (A) SOCS3 promoter constructs (-2000/-1) co-transfected with pcDNA3.1-EYA2 or/and GV141-DACH1 and the relative luciferase activity measured in MHCC-97H cell line. (B) Western blot analysis of expression of SOCS3, p-STAT3, p-JAK2, STAT3 and JAK2 in MHCC-97H cells co-transfected with si-DACH1/pcDNA3.1-EYA2. (C) Western blot analysis of expression of SOCS3 in MHCC-97H and Huh-7 cells infected with lentivirus-EYA2 and lentivirus-EYA2(A510E) mutant. **P* < 0.05. (A, B) mean ± SEM, Student’s *t*-tests.

**Figure S12.** Detection of *Eya2*^-/-^ and *Eya2*^+/+^ mice of non-induced group at 11 months. (A) DNA electrophoretic map of genotype identification of *Eya2*^-/-^ mice. Electrophoresis of the PCR products of the Alb-Cre gene (left) and EYA2 (right). *Eya2*^+/+^ represented wild-type mouse with a band size of 278 bp and *Eya2*^-/-^ represented hepatocyte-specific EYA2 knockout mouse with a band size of 358 bp. (B) Images of the liver from non-induced *Eya2*^-/-^ and *Eya2*^+/+^ mice. (C) H&E staining and immunohistochemistry analysis of the expression of EYA2, SOCS3 and PCNA in liver tissues from the non-induced mice.

**Figure S13.** The level of STAT3 phosphorylation in response to IL-6 in *Eya2*^-/-^ hepatocytes. STAT3 phosphorylation induced by IL-6 was significantly higher in EYA2-deficient hepatocytes than control littermates at each point of time.
