# Supplementary material for: EYA2 suppresses the progression of hepatocellular carcinoma via SOCS3-mediated blockade of JAK/STAT signaling
Source: Mol Cancer. 2021 May 27;20:79. doi: 10.1186/s12943-021-01377-9 (PMC8157759; doi:10.1186/s12943-021-01377-9)
Supplement: Supplementary file 3 — Additional file 3: Fig. S1. The classification and number of somatic mutations identified by whole exome sequencing. Fig. S2. Somatic mutations identified in candidate gene GPR98, EYA2 and UBE2S, which were confirmed by IGV visualization from HCC and case-matched adjacent tissues. The bold numbers in the figures represent allelic fraction of the altered base. Fig. S3. The mRNA expression and function of EYA2 in pan-cancer. (A) mRNA expression of EYA2 in pan-cancer and their respective adjacent based on TCGA database. (B) mRNA expression of EYA2 in pan-cancer cells based on CCLE database. (C) Western blot analysis of the expression of EYA2 in four cancer cells transiently transfected with overexpression vectors. (D) The effect of pcDNA3.1-mediated overexpression of constructs encoding EYA2 wild-type on cell proliferation of cancer cells in vitro. (E) The effect of EYA2 wild-type on cell invasion of cancer cells in vitro. ACC, adrenocortical carcinoma; BLCA, bladder urothelial carcinoma; BRCA, breast invasive carcinoma; CESC, cervical squamous cell carcinoma and endocervical adenocarcinoma; CHOL, cholangiocarcinoma; COAD, colon adenocarcinoma; DLBC, lymphoid neoplasm diffuse large B-cell lymphoma; ESCA, esophageal carcinoma; GBM, glioblastoma multiforme; HNSC, head and neck squamous cell carcinoma; KICH, kidney chromophobe; KIRC, kidney renal clear cell carcinoma; KIRP, kidney renal papillary cell carcinoma; LAML, acute myeloid leukemia; LGG, brain lower grade glioma; LIHC, liver hepatocellular carcinoma; LUAD, lung adenocarcinoma; LUSC, lung squamous cell carcinoma; MESO, mesothelioma; OV, ovarian serous cystadenocarcinoma; PAAD, pancreatic adenocarcinoma; PCPG, pheochromocytoma and paraganglioma; PRAD, prostate adenocarcinoma; READ, rectum adenocarcinoma; SARC, sarcoma; SKCM, skin cutaneous melanoma; STAD, stomach adenocarcinoma; TGCT, testicular germ cell tumors; THCA, thyroid carcinoma; THYM, thymoma; UCEC, uterine corpus endometrial carcinoma; UCS, uterine carcin [file 12943_2021_1377_MOESM3_ESM.pdf]

Figure S1

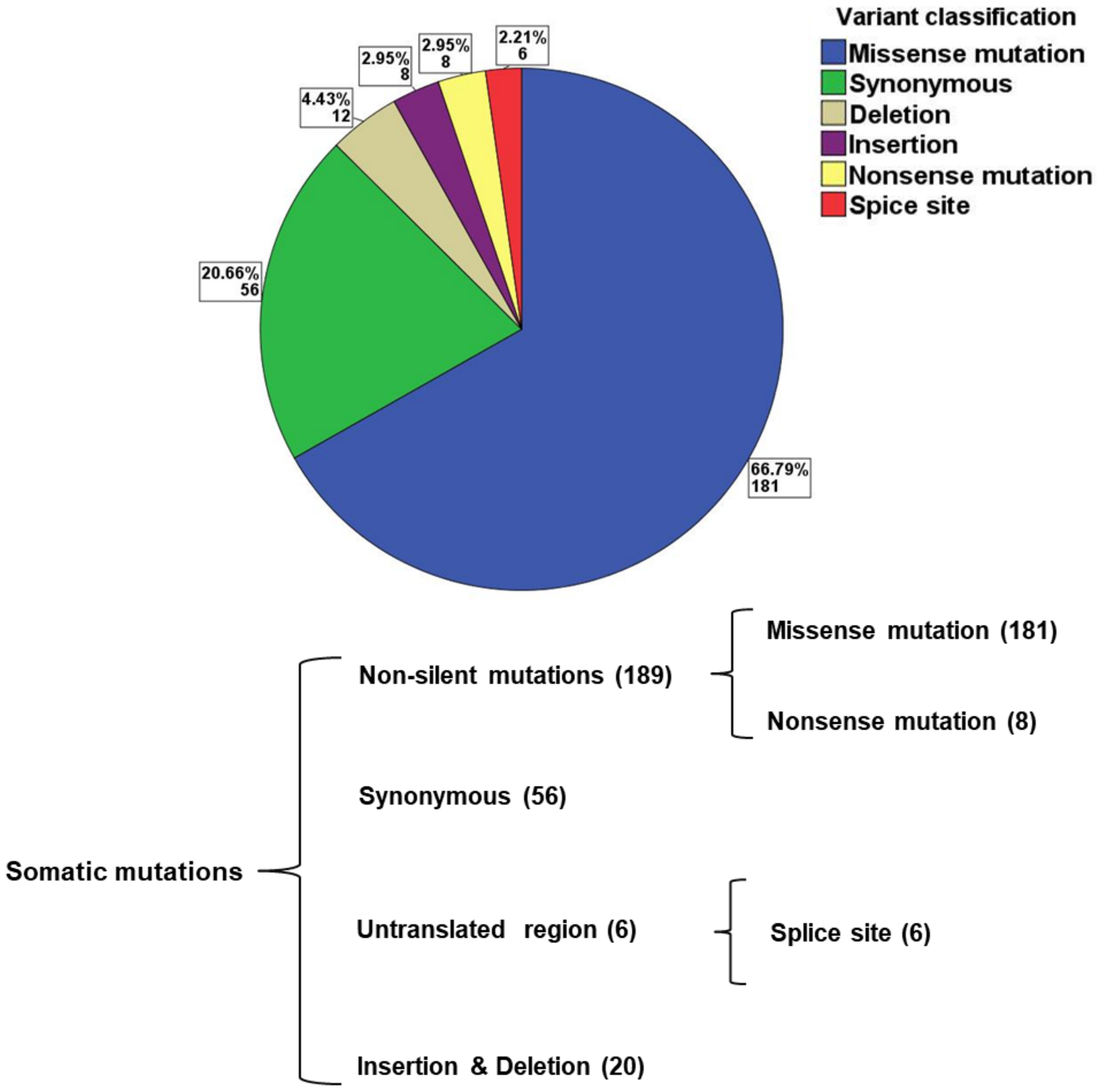
Variant classification
Missense mutation
Synonymous
Deletion
Insertion
Nonsense mutation
Spice site
2.95% 8
2.95% 8
2.21% 6
4.43% 12
20.66% 56
66.79% 181
Somatic mutations
Non-silent mutations (189)
Missense mutation (181)
Nonsense mutation (8)
Synonymous (56)
Untranslated region (6)
Splice site (6)
Insertion & Deletion (20)

Figure S2

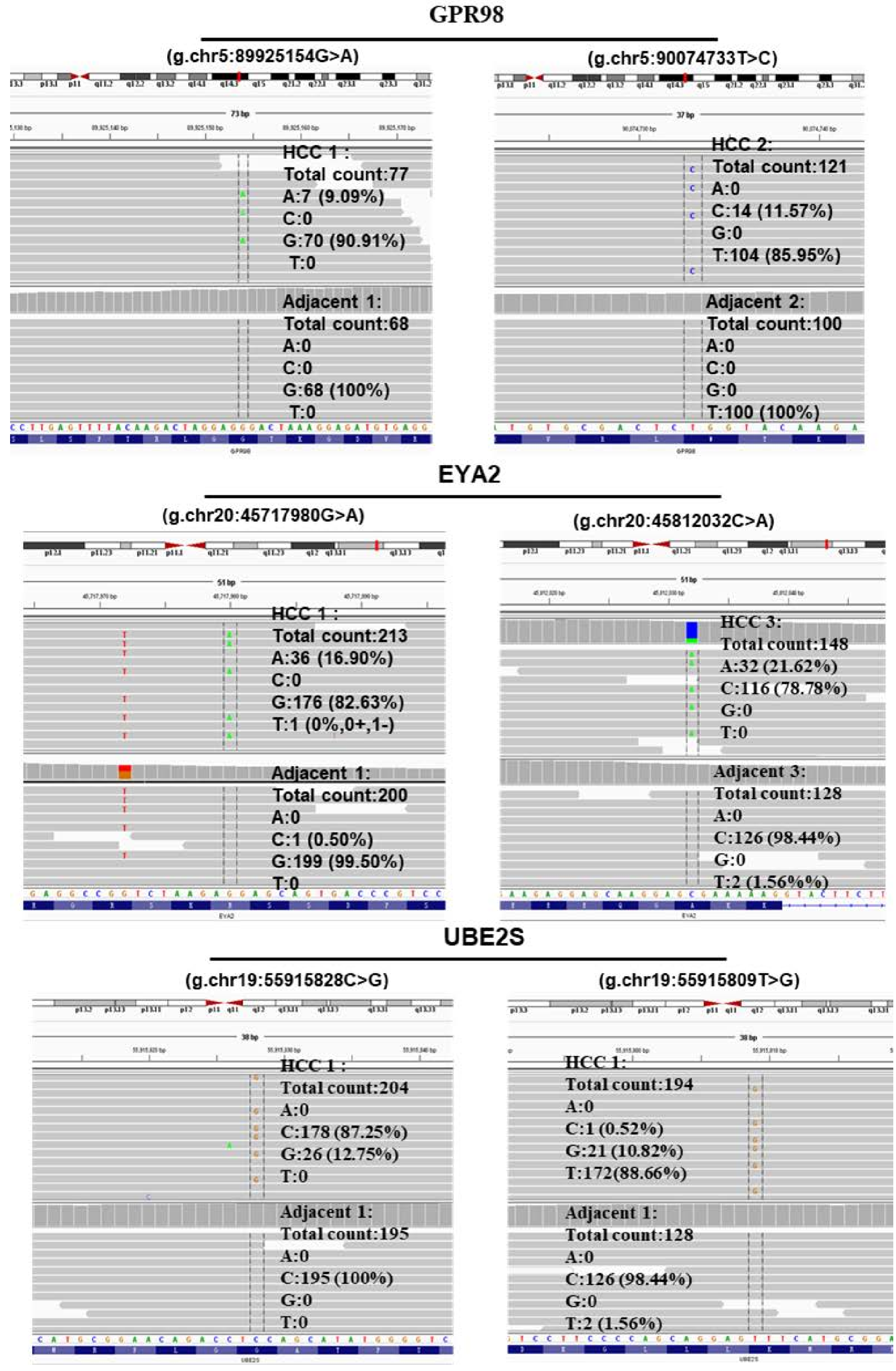

GPR98
(g.chr5:89925154G>A)
HCC 1 :
Total count:77
A:7 (9.09%)
C:0
G:70 (90.91%)
T:0
Adjacent 1:
Total count:68
A:0
C:0
G:68 (100%)
T:0
(g.chr5:90074733T>C)
HCC 2:
Total count:121
A:0
C:14 (11.57%)
G:0
T:104 (85.95%)
Adjacent 2:
Total count:100
A:0
C:0
G:0
T:100 (100%)
EYA2
(g.chr20:45717980G>A)
HCC 1 :
Total count:213
A:36 (16.90%)
C:0
G:176 (82.63%)
T:1 (0%,0+,1-)
Adjacent 1:
Total count:200
A:0
C:1 (0.50%)
G:199 (99.50%)
T:0
(g.chr20:45812032C>A)
HCC 3:
Total count:148
A:32 (21.62%)
C:116 (78.78%)
G:0
T:0
Adjacent 3:
Total count:128
A:0
C:126 (98.44%)
G:0
T:2 (1.56%%)
UBE2S
(g.chr19:55915828C>G)
HCC 1 :
Total count:204
A:0
C:178 (87.25%)
G:26 (12.75%)
T:0
Adjacent 1:
Total count:195
A:0
C:195 (100%)
G:0
T:0
(g.chr19:55915809T>G)
HCC 1:
Total count:194
A:0
C:1 (0.52%)
G:21 (10.82%)
T:172(88.66%)
Adjacent 1:
Total count:128
A:0
C:126 (98.44%)
G:0
T:2 (1.56%)

Figure S3

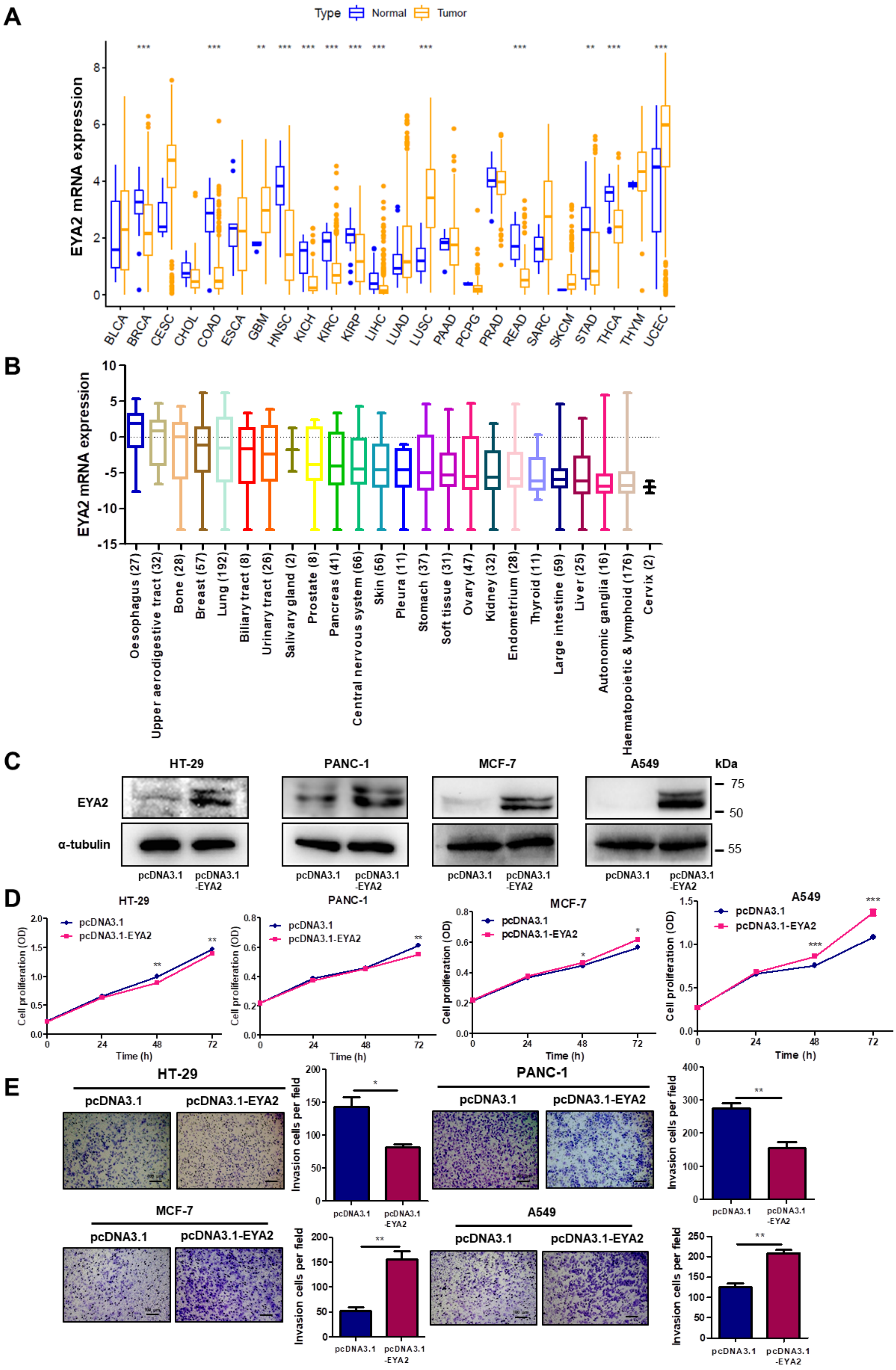
A
Type
Normal
Tumor
EYA2 mRNA expression
***
**
BLCA
BRCA
CESC
CHOL
COAD
ESCA
GBM
HNSC
KICH
KIRC
KIRP
LIHC
LUAD
LUSC
PAAD
PCPG
PRAD
READ
SARC
SKCM
STAD
THCA
THYM
UCEC
B
EYA2 mRNA expression
Oesophagus (27)
Upper aerodigestive tract (32)
Bone (28)
Breast (57)
Lung (192)
Biliary tract (8)
Urinary tract (26)
Salivary gland (2)
Prostate (8)
Pancreas (41)
Central nervous system (66)
Skin (56)
Pleura (11)
Stomach (37)
Soft tissue (31)
Ovary (47)
Kidney (32)
Endometrium (28)
Thyroid (11)
Large intestine (59)
Liver (25)
Autonomic ganglia (16)
Haematopoietic & lymphoid (176)
Cervix (2)
C
HT-29
PANC-1
MCF-7
A549
kDa
EYA2
α-tubulin
75
50
55
pcDNA3.1
pcDNA3.1 -EYA2
D
HT-29
PANC-1
MCF-7
A549
pcDNA3.1
pcDNA3.1-EYA2
Cell proliferation (OD)
Time (h)
E
HT-29
PANC-1
MCF-7
A549
pcDNA3.1
pcDNA3.1-EYA2
Invasion cells per field

Figure S4

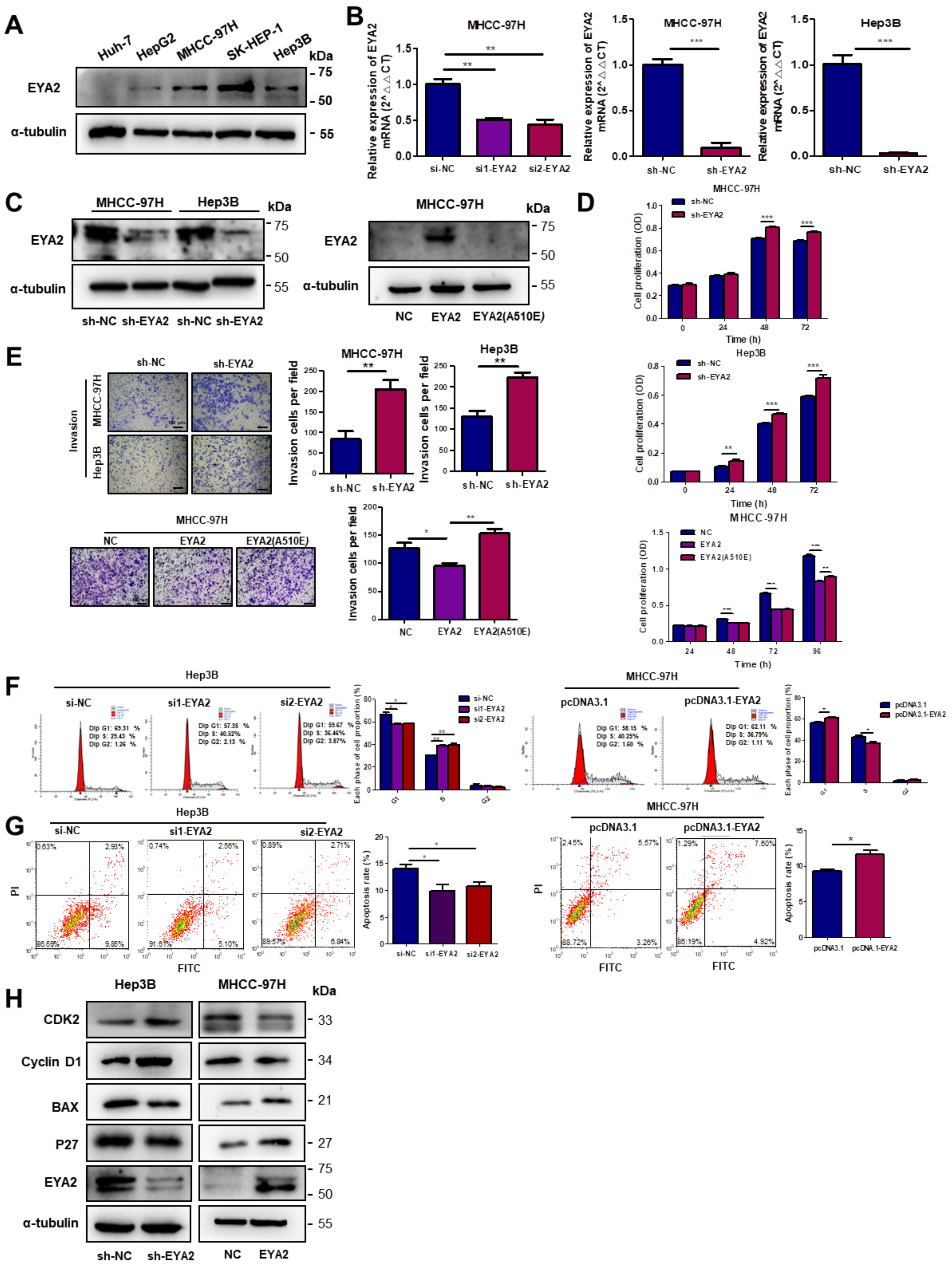

A
Huh-7
HepG2
MHCC-97H
SK-HEP-1
Hep3B
kDa
EYA2
α-tubulin
B
MHCC-97H
Hep3B
Relative expression of EYA2 mRNA (2^△△CT)
si-NC
si1-EYA2
si2-EYA2
sh-NC
sh-EYA2
C
EYA2
α-tubulin
NC
EYA2
EYA2(A510E)
D
Cell proliferation (OD)
Time (h)
E
Invasion
Invasion cells per field
F
Each phase of cell proportion (%)
pcDNA3.1
pcDNA3.1-EYA2
G
PI
FITC
Apoptosis rate (%)
H
CDK2
Cyclin D1
BAX
P27

Figure S5

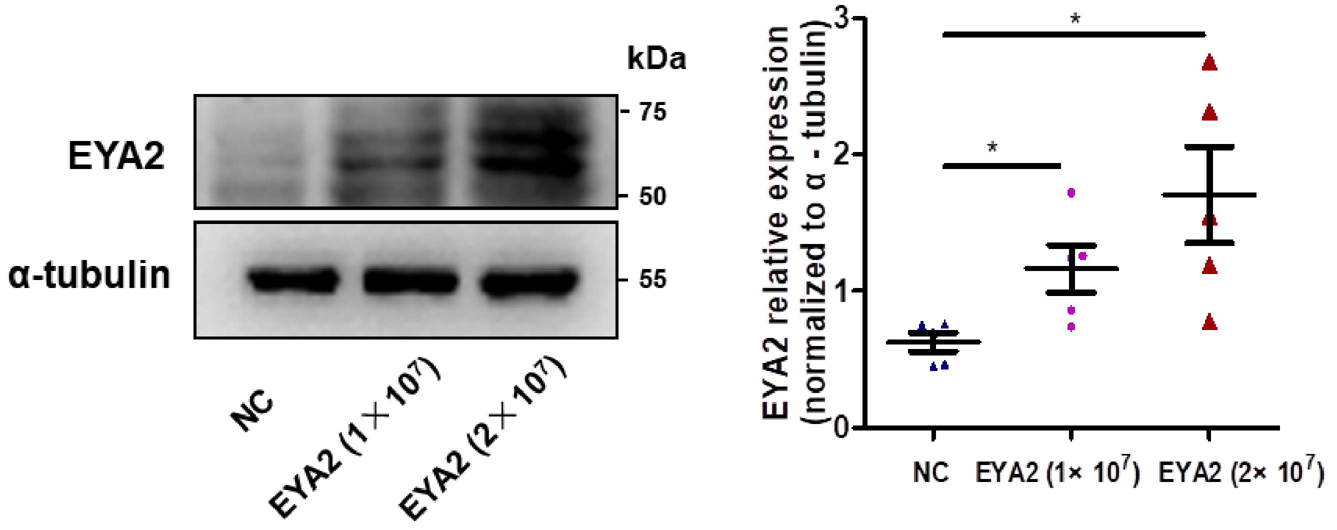
kDa
EYA2
α-tubulin
75
50
55
NC
EYA2 (1×10⁷)
EYA2 (2×10⁷)
EYA2 relative expression
(normalized to α - tubulin)
3
2
1
0
*
*
NC
EYA2 (1× 10⁷)
EYA2 (2× 10⁷)

Figure S6

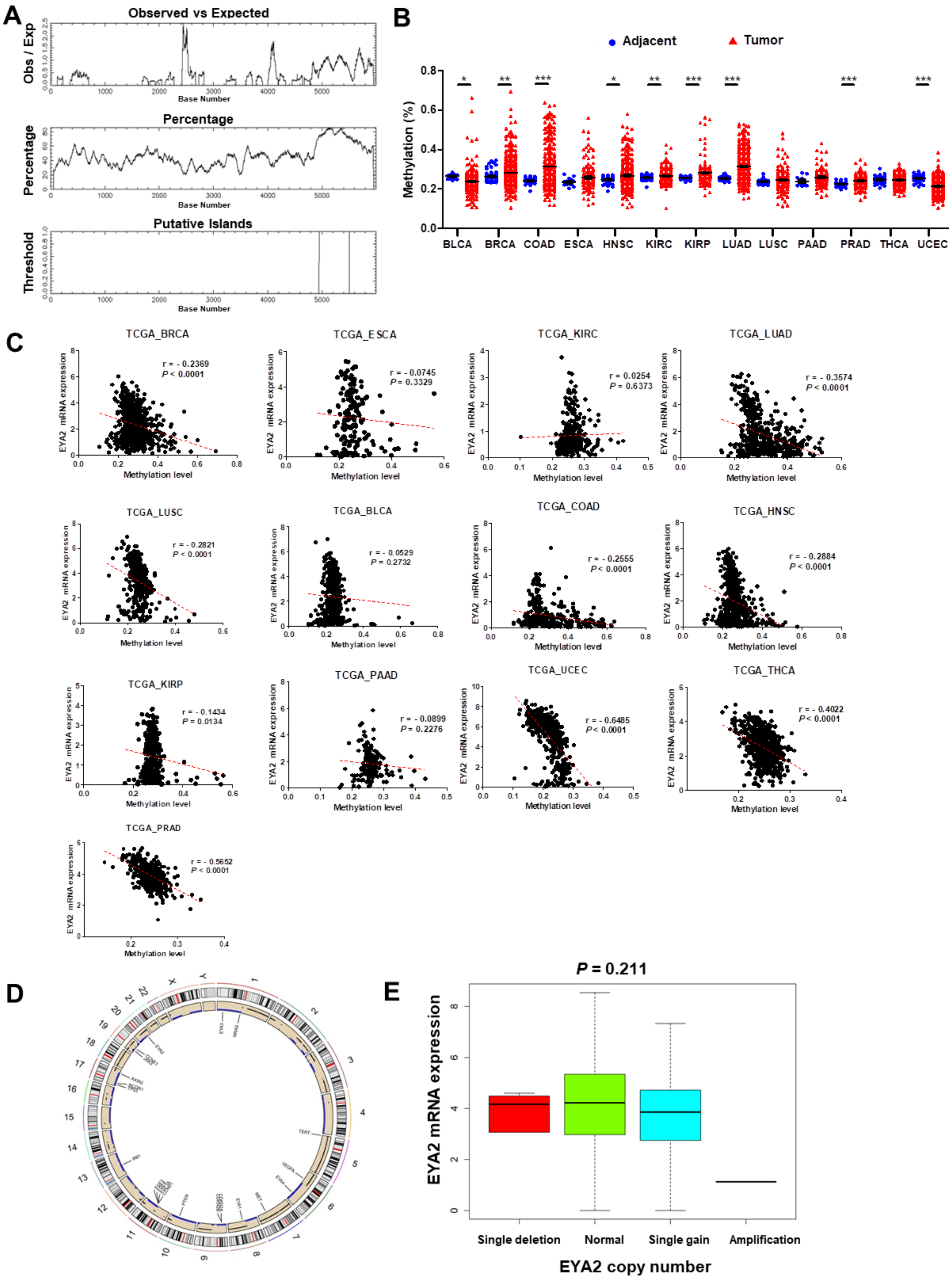
A
Observed vs Expected
Obs / Exp
Base Number
Percentage
Percentage
Base Number
Putative Islands
Threshold
Base Number
B
Adjacent
Tumor
Methylation (%)
BLCA
BRCA
COAD
ESCA
HNSC
KIRC
KIRP
LUAD
LUSC
PAAD
PRAD
THCA
UCEC
C
TCGA_BRCA
r = - 0.2369
P < 0.0001
TCGA_ESCA
r = - 0.0745
P = 0.3329
TCGA_KIRC
r = 0.0254
P = 0.6373
TCGA_LUAD
r = - 0.3574
P < 0.0001
TCGA_LUSC
r = - 0.2821
P < 0.0001
TCGA_BLCA
r = - 0.0529
P = 0.2732
TCGA_COAD
r = - 0.2555
P < 0.0001
TCGA_HNSC
r = - 0.2884
P < 0.0001
TCGA_KIRP
r = - 0.1434
P = 0.0134
TCGA_PAAD
r = - 0.0899
P = 0.2276
TCGA_UCEC
r = - 0.6485
P < 0.0001
TCGA_THCA
r = - 0.4022
P < 0.0001
TCGA_PRAD
r = - 0.5652
P < 0.0001
EYA2 mRNA expression
Methylation level
D
E
P = 0.211
EYA2 mRNA expression
Single deletion
Normal
Single gain
Amplification
EYA2 copy number

Figure S7

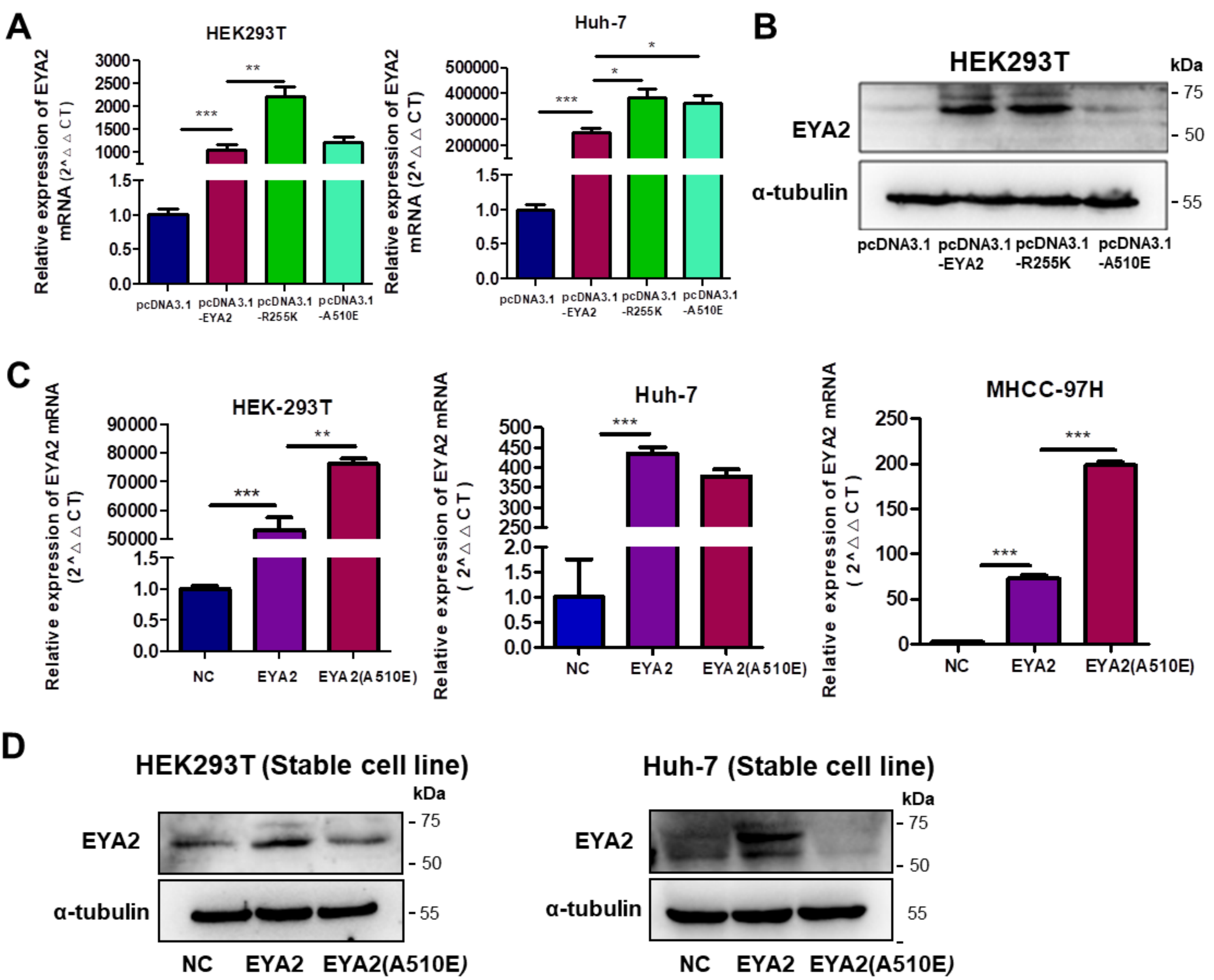
A
HEK293T
Huh-7
Relative expression of EYA2 mRNA (2^△△CT)
pcDNA3.1
pcDNA3.1 -EYA2
pcDNA3.1 -R255K
pcDNA3.1 -A510E
B
HEK293T
kDa
EYA2
α-tubulin
C
HEK-293T
Huh-7
MHCC-97H
Relative expression of EYA2 mRNA (2^△△CT)
NC
EYA2
EYA2(A510E)
D
HEK293T (Stable cell line)
Huh-7 (Stable cell line)
kDa
EYA2
α-tubulin
NC
EYA2
EYA2(A510E)

Figure S8

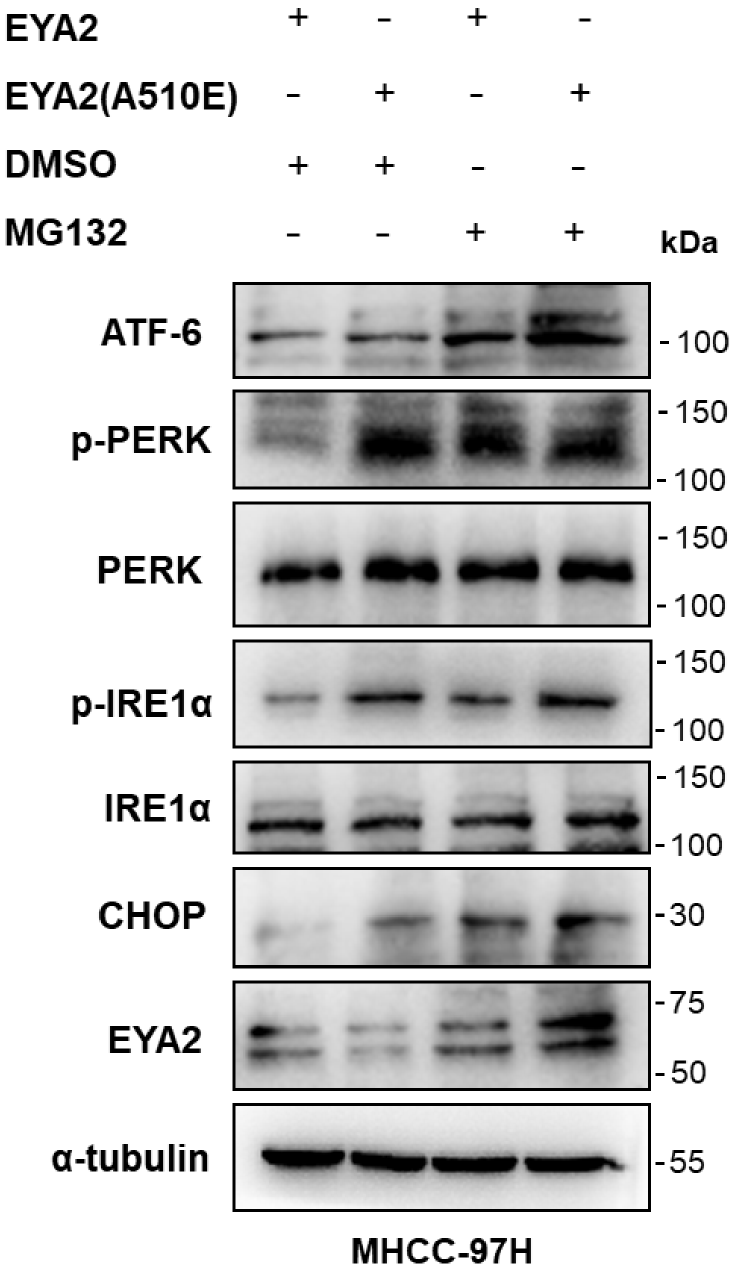
EYA2 + - + -
EYA2(A510E) - + - +
DMSO + + - -
MG132 - - + +
kDa
ATF-6
100
p-PERK
150
100
PERK
150
100
p-IRE1α
150
100
IRE1α
150
100
CHOP
30
EYA2
75
50
α-tubulin
55
MHCC-97H

Figure S9

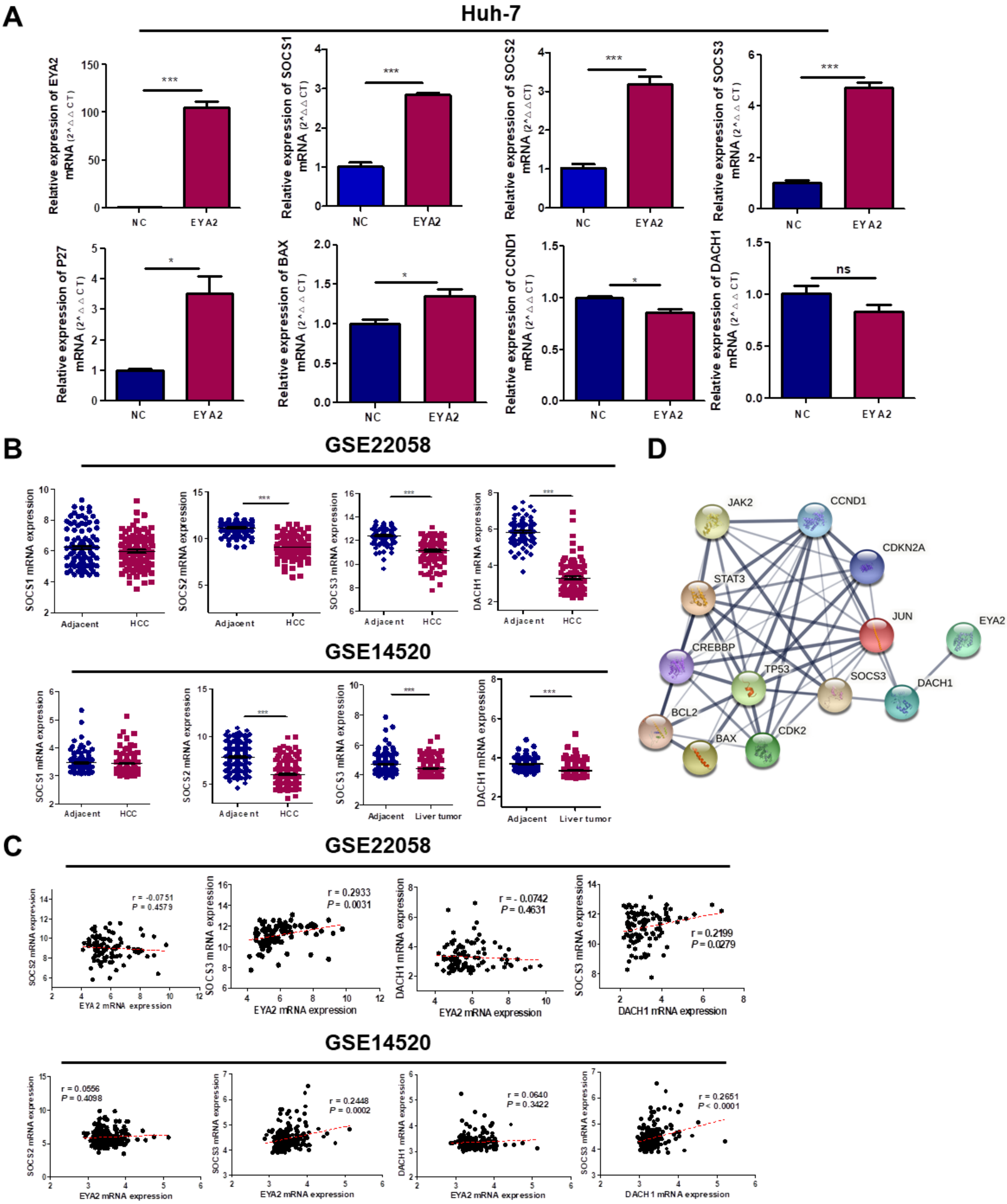
A
Huh-7
Relative expression of EYA2 mRNA (2^△△CT)
Relative expression of SOCS1 mRNA (2^△△CT)
Relative expression of SOCS2 mRNA (2^△△CT)
Relative expression of SOCS3 mRNA (2^△△CT)
Relative expression of P27 mRNA (2^△△CT)
Relative expression of BAX mRNA (2^△△CT)
Relative expression of CCND1 mRNA (2^△△CT)
Relative expression of DACH1 mRNA (2^△△CT)
NC
EYA2
***
*
ns
B
GSE22058
SOCS1 mRNA expression
SOCS2 mRNA expression
SOCS3 mRNA expression
DACH1 mRNA expression
Adjacent
HCC
GSE14520
Liver tumor
C
GSE22058
EYA2 mRNA expression
DACH1 mRNA expression
r = -0.0751
P = 0.4579
r = 0.2933
P = 0.0031
r = - 0.0742
P = 0.4631
r = 0.2199
P = 0.0279
GSE14520
r = 0.0556
P = 0.4098
r = 0.2448
P = 0.0002
r = 0.0640
P = 0.3422
r = 0.2651
P < 0.0001
D
JAK2
CCND1
CDKN2A
STAT3
JUN
EYA2
CREBBP
TP53
SOCS3
DACH1
BCL2
BAX
CDK2

Figure S10

A

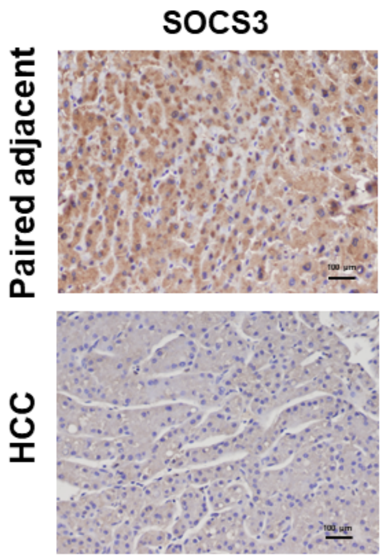

B

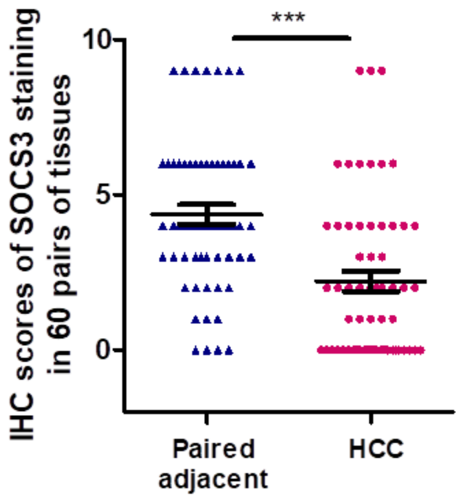

C

	EYA2			r	*P*
	Low	High	Total		
SOCS3				0.386	0.002
Low	33	9	42		
High	7	11	18		
Total	40	20	60		

D

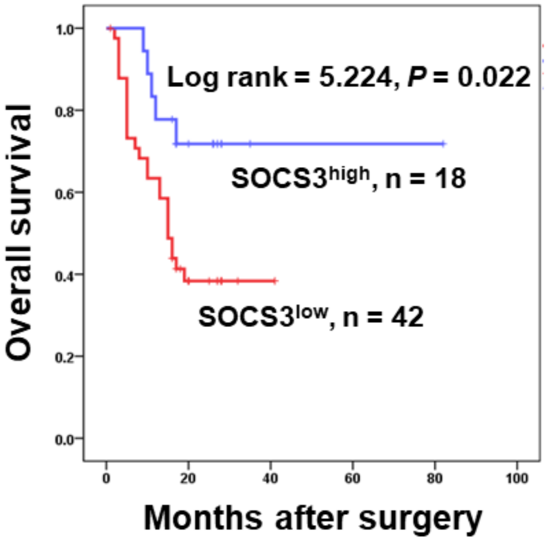

Figure S11

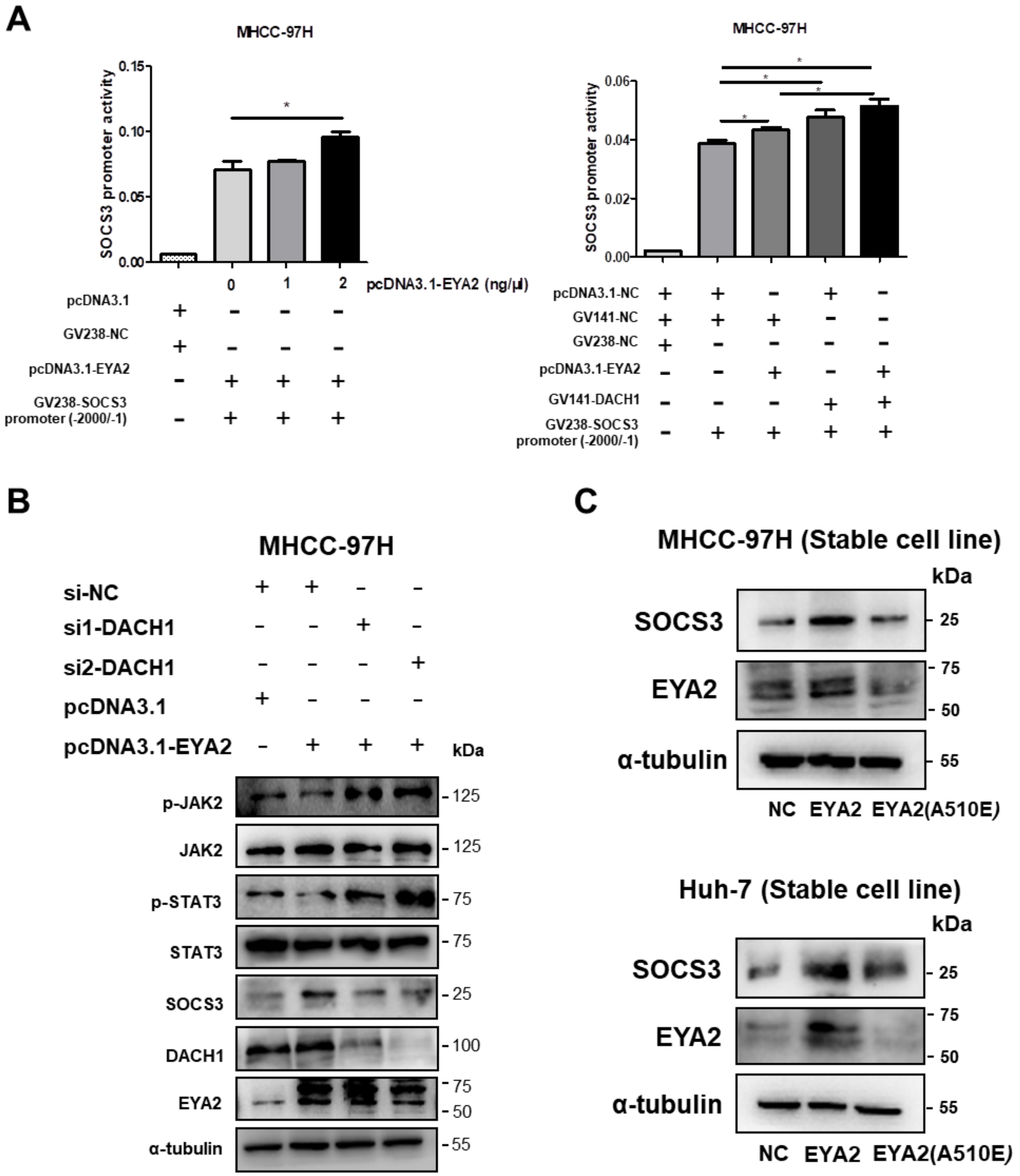
A
MHCC-97H
SOCS3 promoter activity
0.15
0.10
0.05
0.00
0
1
2
pcDNA3.1-EYA2 (ng/μl)
pcDNA3.1
GV238-NC
pcDNA3.1-EYA2
GV238-SOCS3 promoter (-2000/-1)
MHCC-97H
SOCS3 promoter activity
0.06
0.04
0.02
0.00
pcDNA3.1-NC
GV141-NC
GV238-NC
pcDNA3.1-EYA2
GV141-DACH1
GV238-SOCS3 promoter (-2000/-1)
B
MHCC-97H
si-NC
si1-DACH1
si2-DACH1
pcDNA3.1
pcDNA3.1-EYA2
kDa
p-JAK2
JAK2
p-STAT3
STAT3
SOCS3
DACH1
EYA2
α-tubulin
125
125
75
75
25
100
75
50
55
C
MHCC-97H (Stable cell line)
kDa
SOCS3
EYA2
α-tubulin
25
75
50
55
NC
EYA2
EYA2(A510E)
Huh-7 (Stable cell line)
kDa
SOCS3
EYA2
α-tubulin
25
75
50
55
NC
EYA2
EYA2(A510E)

Figure S12

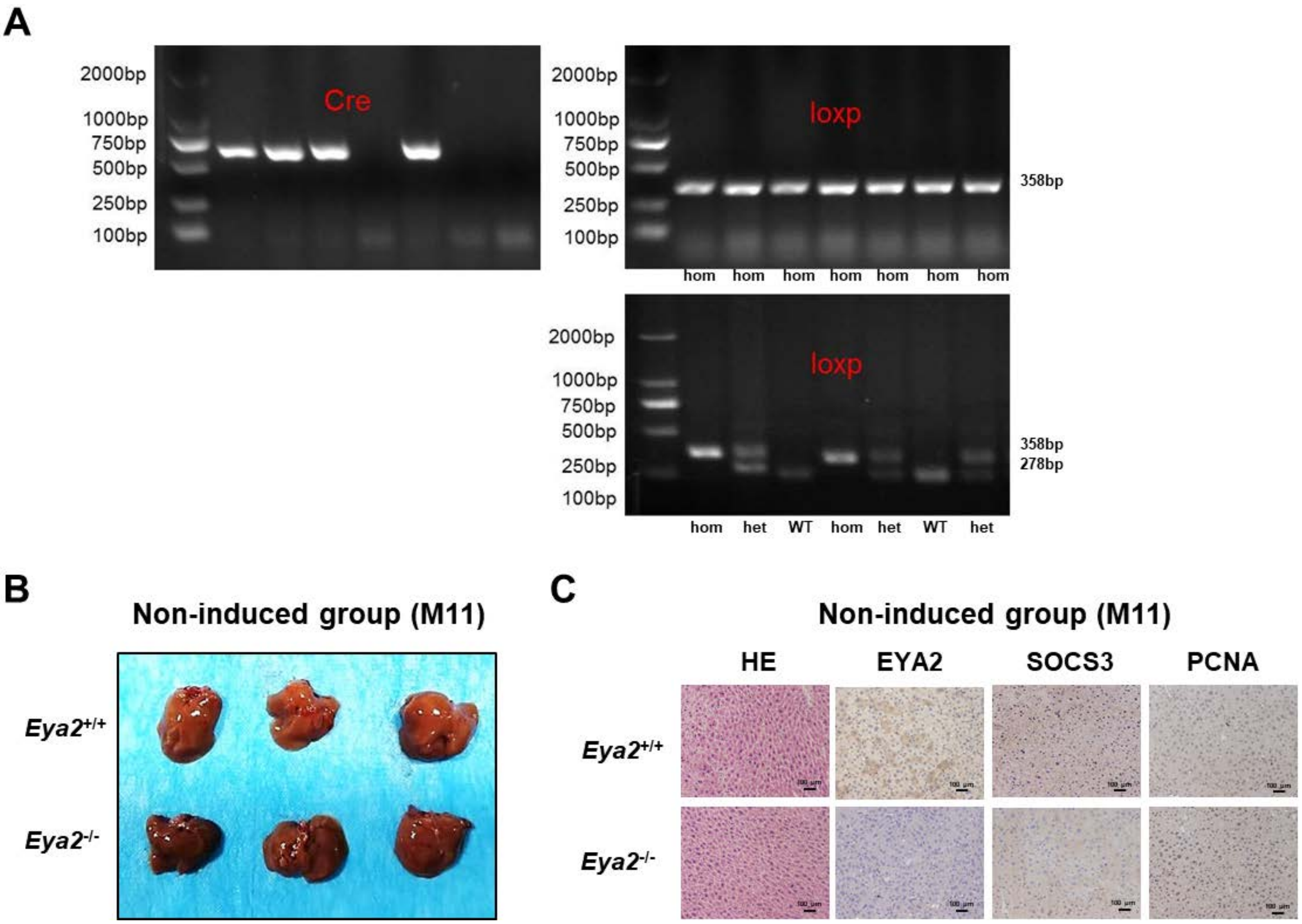
A
2000bp
1000bp
750bp
500bp
250bp
100bp
Cre
loxp
358bp
hom hom hom hom hom hom hom
loxp
358bp
278bp
hom het WT hom het WT het
B
Non-induced group (M11)
Eya2+/+
Eya2-/-
C
Non-induced group (M11)
HE
EYA2
SOCS3
PCNA
Eya2+/+
Eya2-/-

Figure S13

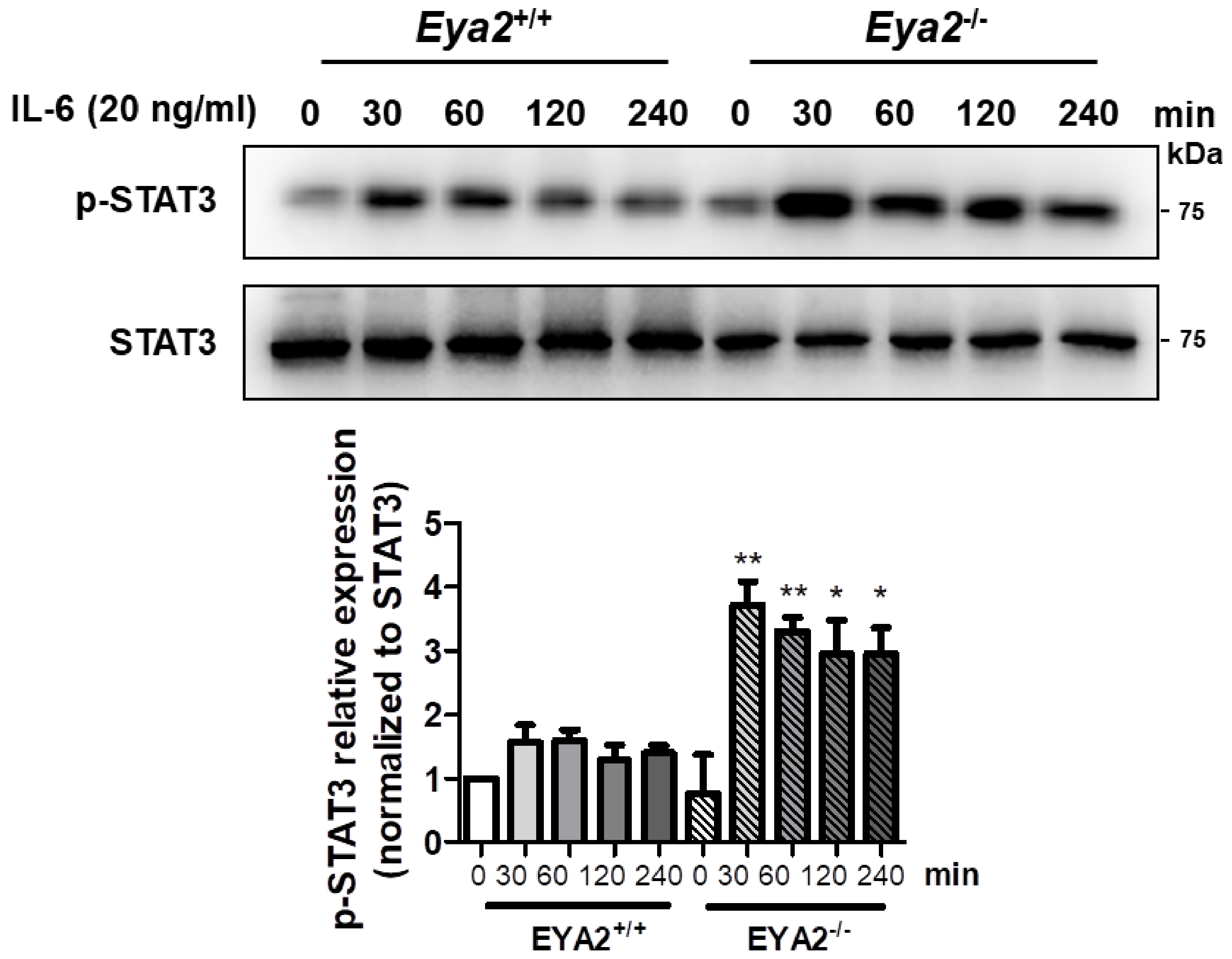

Eya2+/+
Eya2-/-
IL-6 (20 ng/ml)
0 30 60 120 240 0 30 60 120 240 min
kDa
p-STAT3
75
STAT3
75
p-STAT3 relative expression (normalized to STAT3)
5
4
3
2
1
0
**
**
*
*
0 30 60 120 240 0 30 60 120 240 min
EYA2+/+
EYA2-/-
